# Cardiovascular and neuropsychiatric risks of varenicline: a retrospective cohort study

**DOI:** 10.1016/S2213-2600(15)00320-3

**Published:** 2015-10

**Authors:** Daniel Kotz, Wolfgang Viechtbauer, Colin Simpson, Onno C P van Schayck, Robert West, Aziz Sheikh

**Affiliations:** a Institute of General Practice, Medical Faculty of the Heinrich-Heine-University Düsseldorf, Düsseldorf, Germany; b Department of Family Medicine, CAPHRI School for Public Health and Primary Care, Maastricht University Medical Centre, Maastricht, Netherlands; c Allergy and Respiratory Research Group, Centre for Medical Informatics, Usher Institute of Population Health Sciences and Informatics, University of Edinburgh, Edinburgh, UK; d Cancer Research UK Health Behaviour Research Centre, University College London, London, UK; e MHeNS School for Mental Health and Neuroscience, Maastricht University, Maastricht, Netherlands; f Division of General Internal Medicine and Primary Care, Brigham and Women's Hospital, Harvard Medical School, Boston, MA, USA

## Abstract

**Background:**

Varenicline is an effective pharmacotherapy to aid smoking cessation. However, its use is limited by continuing concerns about possible associated risks of serious adverse cardiovascular and neuropsychiatric events. The aim of this study was to investigate whether use of varenicline is associated with such events.

**Methods:**

In this retrospective cohort study, we used data from patients included in the validated QResearch database, which holds data from 753 National Health Service general practices across England. We identified patients aged 18–100 years (registered for longer than 12 months before data extraction) who received a prescription of nicotine replacement treatment (NRT; reference group), bupropion, or varenicline. We excluded patients if they had used one of the drugs during the 12 months before the start date of the study, had received a prescription of a combination of these drugs during the follow-up period, or were temporary residents. We followed patients up for 6 months to compare incident cardiovascular (ischaemic heart disease, cerebral infarction, heart failure, peripheral vascular disease, and cardiac arrhythmia) and neuropsychiatric (depression and self-harm) events using Cox proportional hazards models, adjusted for potential confounders (primary outcomes).

**Findings:**

We identified 164 766 patients who received a prescription (106 759 for nicotine replacement treatment; 6557 for bupropion; 51 450 for varenicline) between Jan 1, 2007, and June 30, 2012. Neither bupropion nor varenicline showed an increased risk of any cardiovascular or neuropsychiatric event compared with NRT (all hazard ratios [HRs] less than 1. Varenicline was associated with a significantly reduced risk of ischaemic heart disease (HR 0·80 [95%CI 0·72–0·87]), cerebral infarction (0·62 [0·52–0·73]), heart failure (0·61 [0·45–0·83]), arrhythmia (0·73 [0·60–0·88]), depression (0·66 [0·63–0·69]), and self-harm (0·56 [0·46–0·68]).

**Interpretation:**

Varenicline does not seem to be associated with an increased risk of documented cardiovascular events, depression, or self-harm when compared with NRT. Adverse events that do not come to attention of general practitioners cannot be excluded. These findings suggest an opportunity for physicians to prescribe varenicline more broadly, even for patients with comorbidities, thereby helping more smokers to quit successfully than do at present.

**Funding:**

Egton Medical Information Systems, University of Nottingham, Ministry of Innovation, Science and Research of the German Federal State of North Rhine-Westphalia, Cancer Research UK, Medical Research Council, Commonwealth Fund.

## Introduction

Cigarette smoking continues to be one of the leading causes of preventable death, killing nearly 6 million people worldwide each year.^1^ Smokers who do not stop lose, on average, a decade of life expectancy.^2^ Effective pharmacotherapies to help smokers quit include bupropion, nicotine replacement treatment (NRT), and varenicline.^3–5^ Varenicline, a selective α4β2 nicotine acetylcholine receptor partial agonist, was approved by the US Food and Drug Administration (FDA) and European Medicines Agency as a drug for smoking cessation in 2006, and it has subsequently been recommended by US^6^ and international^7,8^ clinical guidelines.^6–8^ Varenicline is more effective than are bupropion^5,9–11^ and single forms of NRT,^9,12,13^ and it has become the most frequently prescribed smoking cessation drug other than NRT.^14^ It is also effective and safe in increasing long-term smoking cessation rates via smoking reduction in cigarette smokers not willing or able to quit at treatment initiation.^15^

The safety profile of varenicline was initially established with standard approaches to pharmacovigilance. However, subsequent postmarketing reports raised concerns about the risk of serious adverse cardiovascular and neuropsychiatric events. Authors of a meta-analysis^16^ reported an increased risk of cardiovascular events in varenicline users. Although this study had some major limitations,^17^ and later meta-analyses did not find a significant association,^9,18^ cardiovascular events have been included by the FDA as a warning in the drug's prescribing information.^19^ Possible mechanisms for increased cardiovascular risk could relate to varenicline's action on α3β4 receptors in the peripheral ganglia and subsequent release of acetylcholine and catecholamines, and the central effect of α4β2 and α7 nicotinic acetylcholine receptors on blood pressure homoeostasis.^20^

**Research in context**

**Evidence before this study**

We searched PubMed and the Food and Drug Administration website for relevant reports, and the reference lists of the identified reports. Authors of one meta-analysis reported a small but significantly increased risk of a composite of cardiovascular events in varenicline users, but authors of two later meta-analyses did not. Investigators of a retrospective cohort study with use of a Danish patient registry noted neither a significant increase in a composite of cardiovascular events nor an increase in acute coronary syndrome, ischaemic stroke, or cardiovascular death. Investigators of two retrospective cohort studies with use of data from a UK general practice database did not find an increased risk of depression, suicidal thoughts, or self-harm in varenicline compared with nicotine replacement treatment (NRT) users. Investigators of a retrospective cohort study with use of a US military health system claims database did not find an increase in the proportion of neuropsychiatric admissions to hospital in patients given varenicline compared with NRT patches.

**Added value of this study**

This study is, to our knowledge, the largest study ever done on this topic. It uses a validated general practice dataset in a country (England) with a national health-care system in which all members of the community have free and ready access to smoking cessation treatment. It includes, to our knowledge for the first time, the most important neuropsychiatric and cardiovascular adverse events in one and the same study. It uses propensity score matching and regression modelling to take maximum account of confounding. Finally, it models what would need to be the distribution and effect of unmeasured confounders for the key conclusions to be incorrect.

**Implications of all the available evidence**

The findings from our study substantiate the results of meta-analyses and previous small observational studies that show that varenicline is not likely to increase the risk of self-harm or depression or any of a wide range of cardiovascular outcomes. Although this study could not rule out adverse reactions that do not get recorded in general practice records, the findings have clear implications for the safety warnings for varenicline and for clinical practice. They suggest an opportunity for physicians to prescribe varenicline more broadly, even for patients with comorbidities, thereby helping more smokers to quit successfully than do at present.

Neuropsychiatric events in varenicline users might, in part, be attributable to smoking itself—ie, to neuropsychiatric disorders that already existed before the quit attempt or to other smoking-related disorders that are themselves associated with increased neuropsychiatric risk.^21,22^ Nevertheless, the FDA and European Medicines Agency issued warnings that serious neuropsychiatric symptoms had occurred in smokers trying to quit with varenicline, consisting of changes in behaviour, agitation, depressed mood, suicidal ideation, and attempted and completed suicides.^23,24^ Moreover, the FDA judged the events identified by the postmarketing reports to be sufficiently indicative of a causal association with the drug to include a black box warning about neuropsychiatric events.

A meta-analysis^18^ of clinical trials, and observational studies^25–30^ carried out in general smoking populations, did not find any increased risk of cardiovascular or neuropsychiatric events in varenicline users. However, this evidence has not been deemed sufficient by the FDA to remove the black box warning or change the label warning.^31^ Previous randomised controlled trials, even when analysed in combination, had limited power to detect rare, serious adverse events, and might have excluded patients who would be most susceptible to experiencing them (because patients need to provide informed consent, and selection of patients occurs through exclusion criteria, resulting in a population that is healthier and less vulnerable than the general population is). Results from previous observational studies have been criticised by the FDA as being biased by residual confounding.^31^

The aims of this study were therefore to investigate the most important cardiovascular and neuropsychiatric adverse events with use of one of the largest validated databases and assess and reduce the risk of confounding further than any previous study has. Our work extends previous studies by being, to our knowledge, the largest study ever done on this topic with use of a general practice dataset in a country (England) with a national health-care system in which all members of the community, irrespective of socioeconomic status, had free and ready access to smoking cessation treatment. To our knowledge, we are the first to include both neuropsychiatric and cardiovascular adverse events in one and the same study and model what the distribution and effect of unmeasured confounders would need to be for the key conclusions to be incorrect.

## Methods

### Study design and patients

In this national, retrospective cohort study, we used the QResearch database (version 36, upload July 31, 2013), which holds anonymised health records for more than 13 million patients from 753 National Health Service general practices from across England. The database has been used for various studies before, including those of the incidence and risk of neuropsychiatric^32^ and cardiovascular^33–36^ events. Findings from external validation studies showed that studies using this database yield similar results to those using other databases, such as the Clinical Practice Research Datalink^37,38^ and Health Improvement Network databases.^39^ The study protocol has been published elsewhere.^40^ The only deviation from our published plan is that we could not do an instrumental variable analysis because we were unable to identify a valid instrumental variable, so we instead did additional analyses (ie, modelling) to assess the effect of any potential unmeasured confounding. Use of this particular method was also prompted by concerns raised by the FDA in relation to evidence from previous observational studies of the safety of varenicline.^31^

We studied adult patients aged 18–100 years (registered for longer than 12 months before data extraction) who received prescriptions for varenicline, bupropion, or NRT between Jan 1, 2007, and June 30, 2012. The date of first prescription of one of these drugs defined the individual's entry date to the cohort. We excluded patients if they had used one of the drugs during the 12 months before the start date of the study (ie, between Jan 1, 2006, and Dec 31, 2006) or received a prescription of a combination of these drugs during the 6 month follow-up period. We also excluded those who were temporary residents.

Our protocol was independently peer reviewed by the QResearch Scientific Board and satisfied the requirements of the Trent Research Ethics Committee.

### Procedures

We classified patients into three exposure groups—varenicline alone, bupropion alone, or NRT alone (used as a reference group because it is presumed by regulators not to carry serious risks)—on the basis of the drug that they were first prescribed. In the UK, all three drugs are only licensed for use to help smoking cessation. Start of follow-up began for each patient on the date of the first prescription and ended after 6 months of follow-up or when reaching the specific event of interest. We censored patients who were lost to follow-up because they left the practice or died.

### Outcomes

We separately considered major incident neuropsychiatric and cardiovascular events that occurred during 6 months of follow-up (on the basis of appropriate Read codes—a coding system used by general practitioners in the UK) for which a potential association with varenicline use has been suggested (primary outcomes).^16,25,26^ The cardiovascular events of interest were ischaemic heart disease (including myocardial infarction and angina), cerebral infarction and haemorrhage, heart failure, peripheral vascular disease, and cardiac arrhythmia (including cardiac arrest). The neuropsychiatric outcomes of interest were depression and fatal or non-fatal intentional self-harm. A follow-up period of 6 months covers the treatment duration of the drugs (typically 12 weeks) and an extended period after treatment is stopped in which many of the spontaneously reported adverse events occurred and the excess in cardiovascular events was noted in meta-analyses of clinical trials. As a secondary outcome, we assessed occurrence of these events during the first 3 months of follow-up.

### Statistical analysis

We used Cox proportional hazards regression models to assess the association between exposure group and each of the events, adjusted for all measured potential confounders. We included the following variables, measured at or before the patient's entry date to the cohort, in the analyses as potential confounders: age, sex, socioeconomic status (measured with the Townsend Index^41^), Strategic Health Authority of the general practice, relevant comorbidities from the Charlson Index^42^ (ie, chronic obstructive pulmonary disease, diabetes, peptic ulcer disease, renal disease, rheumatological disease, or cancer), and alcohol misuse. Additionally, we included any recordings of the neuropsychiatric and cardiovascular events of interest that occurred before the patient's entry date to the cohort.

We entered all variables into the models as binary variables except for the continuous variables age and socioeconomic status. We used a propensity score analysis with trimming and matching to account for potential confounding by indication.^40^ Additionally, we used an approach described by Lin and colleagues^43^ to model the effects of any potential unmeasured confounding. To this end, we adjusted the hazard ratios (HRs) and 95% CIs in varenicline versus NRT users for each of the events for a hypothetical, unmeasured, binary confounder, with an HR of 3 and various combinations of prevalence in the two exposure groups.

We did all analyses in R (version 3.0.2 or later). We provide the codes used in R in the appendix. All statistical tests were two-sided, with p<0·05 showing significance.

### Role of the funding source

The funder of the study provided access to the QResearch database, which included collection and management of data. The funder of the study had no role in study design, data analysis, data interpretation, or writing of the report. The corresponding author had full access to all the data in the study and had final responsibility for the decision to submit for publication.

## Results

We identified 164 766 patients who received prescriptions between Jan 1, 2007, and June 30, 2012, and included them in the analyses: 106 759 NRT users, 6557 bupropion users, and 51 450 varenicline users (figure). A few patients (125 [<1%] of 164 891 patients using NRT, bupropion, or varenicline) had missing data on the measure of socioeconomic status and we therefore excluded them. NRT users were older and more socioeconomically deprived than were bupropion and varenicline users, and showed a higher prevalence of all confounding factors (table 1). We noted the highest incidence of events for depression and ischaemic heart disease (table 2, appendix).

Neither bupropion nor varenicline showed an increased risk of any cardiovascular or neuropsychiatric event compared with NRT (all HRs less than 1·00; primary outcomes; table 2). Rather, varenicline was associated with a significantly reduced risk of ischaemic heart disease (HR 0·80 [95% CI 0·72–0·87), cerebral infarction (0·62 [0·52–0·73]), heart failure (0·61 [95% CI 0·45–0·83]), arrhythmia (0·73 [95% CI 0·60–0·88]), depression (0·66 [0·63–0·69]), and self-harm (0·56 [0·46–0·68]).

χ^2^ tests showed that hazards were not proportional for the outcomes of ischaemic heart disease (p<0·0001), cerebral infarction (p=0·004), and depression (p<0·0001), but a fine-grained analysis allowing for varying HRs showed that HRs were always less than 1 across the entire follow-up period for the three outcomes. Thus, for these three outcomes, the reported HRs can be regarded as an average across the follow-up period. Furthermore, we noted that the risk of arrhythmia (p=0·02) and depression (p<0·0001) in varenicline users compared with NRT differed significantly between women and men, but the HRs were again always less than 1.

After trimming and matching of patients by propensity score, the sample size was 12 786 for the comparison of bupropion with NRT, and 100 326 for that of varenicline with NRT (figure, appendix). Neither bupropion nor varenicline showed any evidence of increased risk of any neuropsychiatric or cardiovascular event compared with NRT (table 3).

The modelling of unmeasured confounding showed that an increased risk of any of the neuropsychiatric and cardiovascular events assessed in varenicline users was very unlikely (appendix). For example, an unmeasured confounder with an HR of 3 for self-harm would have reversed the noted reduced HR in varenicline users versus NRT, making it an increased HR, only if this confounder had been distributed very differently in the two exposure groups. For such an outcome, the prevalence of this confounder would need to be only 10% in varenicline users and simultaneously be noted in at least 80% of NRT users (table 4).

Cox proportional hazards regression and propensity score analyses and modelling of unmeasured confounding with the occurrence of the cardiovascular and neuropsychiatric events during 3 months of follow-up yielded very similar results (secondary outcomes; appendix).

## Discussion

We noted no evidence of any increased risk of cardiovascular or neuropsychiatric adverse events in smokers using varenicline or bupropion when compared with NRT users. On the contrary, some events were associated with a reduced risk, including the events with the highest noted incidences (ie, depression and ischaemic heart disease). We noted that modelling of the effects of any potential unmeasured confounders showed that a confounder would only lead to an increased risk associated with varenicline use under unlikely assumptions.

Authors of one meta-analysis^16^ reported a small but significant increased risk of serious adverse cardiovascular events in varenicline users, whereas authors of two more meta-analyses^9,18^ and a network meta-analysis^44^ did not find an increase in such events. Rapid assessment of cardiovascular outcomes within the FDA's Mini-Sentinel programme showed no consistent evidence of increased cardiovascular risk with varenicline.^45^

Authors of systematic reviews and meta-analyses did not find evidence for an increased risk of serious adverse neuropsychiatric events in varenicline users.^9,46,47^ Authors of two studies^26,27^ with use of data from a UK general practice database similar to the one that we used noted no association between varenicline use and increased risk of depression, suicidal thoughts, or self-harm. In fact, the authors of one of the studies^27^ noted a significantly reduced risk of depression in varenicline (HR 0·75 [95% CI 0·65–0·87]) and bupropion (0·63 [95% CI 0·46–0·87]) users in comparison with NRT; the estimated HRs are similar to ours (but with less precision). Investigators of no other cohort studies^28–30^ noted an association between use of varenicline and neuropsychiatric events.

A striking finding in our study was the difference between patient characteristics at baseline. Compared with bupropion and varenicline users (who had very similar characteristics), NRT users were older and more socioeconomically deprived, and showed a higher prevalence of all of the cardiovascular and neuropsychiatric risk factors and comorbid diseases. In the statistical models used for our analyses, however, these differences were balanced. Investigators of only a few previous observational studies compared NRT with varenicline or bupropion users. Investigators of one study,^28^ done in the US military health system database, did not find a difference in previous neuropsychiatric disease between NRT and varenicline users, whereas investigators of two other studies,^26,27^ both done in the same English general practice database, noted that users of NRT were more likely to have had previous psychiatric events and chronic illness, misuse alcohol, and use hypnotics, antipsychotics, and antidepressants. Thus, varenicline and bupropion seem currently less likely to be prescribed to smokers in general practice with smoking-related illnesses and comorbidities.

Our study has several limitations, most of which are related to the observational study design. In view of the potential importance of unmeasured confounders in observational studies and the large difference in measured confounders between those receiving prescriptions for NRT and those receiving them for bupropion or varenicline, we set out, to our knowledge for the first time, to model whether such confounders could reasonably reverse the study conclusions. We set the combined HR of unmeasured confounders at 3. This HR can be viewed as conservative in the sense that it allows the unmeasured confounders to have a very strong effect (eg, an HR of 3 is equivalent to the increased risk of premature death for present *vs* never smokers^2^). Also, except for previous occurrences of the specific event of interest, none of the noted confounders had an HR as high as 3 in any of the analyses (data not shown). Even so, our results show that the noted reduced risk of neuropsychiatric and cardiovascular events in varenicline versus NRT users could only be reversed, making it an increased risk, if a composite of the unmeasured confounders was distributed very differently in the exposure groups. For any of the events, the prevalence of the unmeasured confounders would need to be at least 20% higher in the NRT group than in the varenicline group for the conclusions to be false (appendix; or 50% higher to generate an HR of 1·5 or higher in varenicline users, a relative hazard that we established as clinically meaningful in our study protocol^40^). None of the noted confounders were distributed so unevenly between the two groups; the most unevenly distributed confounder was previous depression, with a prevalence of 38% in NRT versus 32% in varenicline users (table 1). Thus, our findings are unlikely to be confounded to an extent that would have obscured an increased risk of varenicline. However, the modelling approach as applied has a limitation as well: it assumes that the unmeasured confounder is not associated with other confounders within the exposure group.^43^

A second limitation is that our analyses relied on routinely collected data. Some data might have been incomplete or inaccurate; however, we believe that incomplete or inaccurate data are unlikely in light of the fact that the QResearch database has been validated for answering research questions such as ours.^40^ In most UK general practices, the electronic health record is the only patient record. It is therefore populated mainly for use as a clinical rather than billing record—in many parts of the USA, for example, its main use is as a billing record. Some variables of potential interest were not available, including drug adherence or potential confounders such as previous or present levels of tobacco exposure. For drug adherence, we used general practitioner prescription data, which could overestimate drug exposure because not all patients collect their drugs, and those who do do not always adhere to the prescribed dosing schedule. However, this factor would have biased our results only in the case of systematic differences in drug adherence between the three drugs being studied. Tobacco exposure might be a confounding factor because it is a risk factor for cardiovascular or neuropsychiatric events and might be associated with type of smoking cessation drug.^48^ We attempted to address this issue by including previous cardiovascular and neuropsychiatric events and a range of other smoking-related diseases, recorded at baseline, as potential confounders in our models. However, we had no data for smoking cessation during follow-up to assess potential differences in effectiveness of the three drugs. Thus, we were unable to fully disentangle the complex pathways between type of drug, serious adverse events being studied, and mediating factors of drug adherence and effectiveness in terms of smoking cessation.

We did not measure what the FDA has described as “nuanced” neuropsychiatric symptoms that are difficult to classify or that involve aggression.^49^ Such symptoms probably cannot be addressed with patient records and need specific monitoring in very large randomised controlled trials and observational studies with primary data collection; doubt exists as to whether such studies will ever prove feasible. Nevertheless, the neuropsychiatric symptoms that we used have been identified as some of the most important and are included in the FDA's boxed warning for varenicline, and the high proportion of serious events noted in our study suggests that events of this kind would be unlikely to have not been recorded in the database. Furthermore, in view of the nature of the UK health system, with access to general practitioners being free of charge and, furthermore, them acting as co-ordinators of care, these events are, we believe, very unlikely to be substantially under-recorded. Moreover, under-recording would have only introduced bias if it occurred systematically differently between the three drugs; we have no reason to assume that this systematic difference occurred. A final limitation is that we did not link our dataset to other datasets to assess mortality because fatalities would usually be recorded in this general practitioner dataset within a month.

A major strength of this study is that it is, to our knowledge, the largest original study ever done on this topic. Second, we investigated, with the same methods, five separate cardiovascular and two neuropsychiatric events in one study, of which one—peripheral vascular disease—has not been investigated before, with other cardiovascular events only having been included in meta-analyses as composite outcomes.^16,18^ Third, a major advantage of use of a large general practice database is generalisability of findings compared with randomised controlled trials because almost all individuals living in the UK are registered with a general practice and have free and ready access to smoking cessation treatment, irrespective of their socioeconomic status. A final strength is that we published our protocol in a peer-reviewed journal before we began the analysis.^40^

In this study, we have shown that use of varenicline is very unlikely to be associated with an increased risk of the measured cardiovascular or neuropsychiatric events compared with NRT. Although we cannot rule out adverse reactions that do not get recorded in general practice files, the findings from this study have clear implications for the safety warnings for varenicline and for clinical practice. To not prescribe the most effective smoking cessation drug on any given occasion is likely to lead to substantial loss of life expectancy, even if the patient stops later in life because patients lose, on average, 3 months of life expectancy for each year of continued smoking.^50^ Our findings suggest an opportunity for physicians to prescribe varenicline more broadly, even for patients with comorbidities, and thereby help more smokers to quit successfully than do at present.

## Figures and Tables

**Figure:**
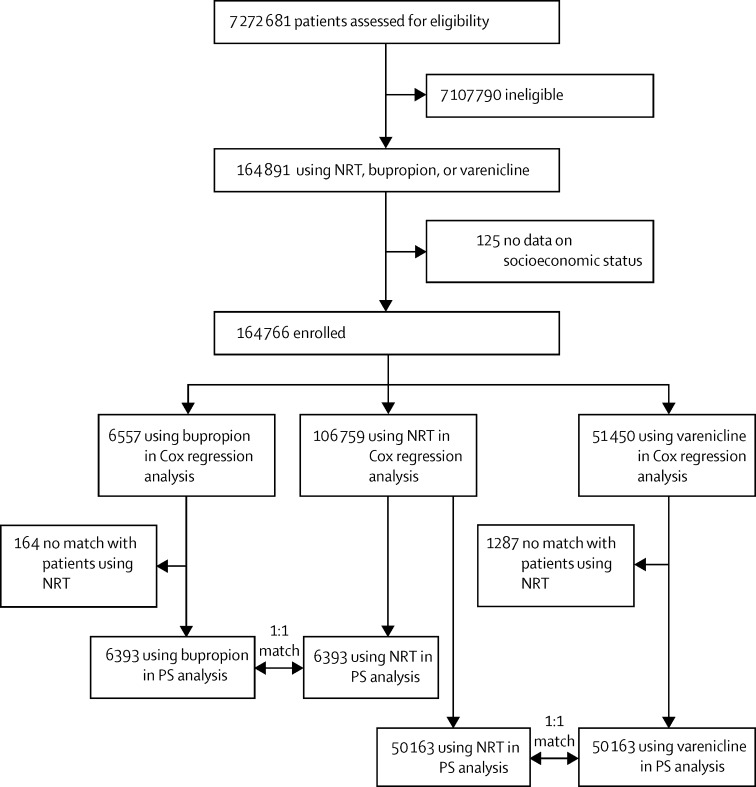
Flow chart NRT=nicotine replacement treatment. PS=propensity score.

**Table 1:** Baseline characteristics

	**NRT (n=106 759)**	**Bupropion (n=6557)**	**Varenicline (n=51 450)**
Age (years)	40·4 (13·6)	37·7 (11·1)	38·1 (11·5)
Female sex	55 345 (52%)	3172 (48%)	24 686 (48%)
Socioeconomic status*	3·2 (1·4)	2·9 (1·3)	3·0 (1·3)
Chronic obstructive pulmonary disease	12 112 (11%)	427 (7%)	4140 (8%)
Diabetes	7415 (7%)	229 (3%)	2511 (5%)
Peptic ulcer disease	3334 (3%)	136 (2%)	1243 (2%)
Renal disease	4618 (4%)	170 (3%)	1457 (3%)
Rheumatological disease	3001 (3%)	106 (2%)	1059 (2%)
Cancer	4311 (4%)	159 (2%)	1449 (3%)
Alcohol misuse	8713 (8%)	321 (5%)	2932 (6%)
Previous ischaemic heart disease	6098 (6%)	175 (3%)	1791 (3%)
Previous cerebral infarction	3477 (3%)	97 (1%)	852 (2%)
Previous heart failure	854 (1%)	19 (<1%)	186 (<1%)
Previous peripheral vascular disease	1354 (1%)	42 (1%)	412 (1%)
Previous arrhythmia	2335 (2%)	85 (1%)	659 (1%)
Previous depression	40 255 (38%)	2215 (34%)	16 242 (32%)
Previous self-harm	12 043 (11%)	610 (9%)	4621 (9%)

Data are mean (SD) or n (%). NRT=nicotine replacement treatment.

* Townsend Index: 1 (lowest level of deprivation) to 5 (highest level of deprivation).^41^

**Table 2:** Incidence of events and hazard ratios of drug groups for all events during 6 month follow-up

	**Patient-years**	**Number of events**	**Incidence of event per 1000 patient-years**	**Hazard ratio**	
				Crude	Adjusted
**Ischaemic heart disease**					
NRT	52 289	2148	41·1	1	1
Bupropion	3246	52	16·0	0·39 (0·30–0·51)	0·67 (0·51–0·89)
Varenicline	25428	594	23·4	0·57 (0·52–0·62)	0·80 (0·72–0·87)
**Cerebral infarction**					
NRT	52 705	871	16·5	1	1
Bupropion	3259	18	5·5	0·33 (0·21–0·53)	0·55 (0·35–0·89)
Varenicline	25 557	164	6·4	0·39 (0·33–0·46)	0·62 (0·52–0·73)
**Heart failure**					
NRT	52 895	302	5·7	1	1
Bupropion	3262	7	2·1	0·38 (0·18–0·80)	0·71 (0·33–1·51)
Varenicline	25 588	52	2·0	0·36 (0·27–0·48)	0·61 (0·45–0·83)
**Peripheral vascular disease**					
NRT	52 849	430	8·1	1	1
Bupropion	3259	14	4·3	0·53 (0·31–0·90)	0·83 (0·48–1·41)
Varenicline	25 563	123	4·8	0·59 (0·48–0·72)	0·82 (0·67–1·01)
**Arrhythmia**					
NRT	52 815	563	10·7	1	1
Bupropion	3260	14	4·3	0·40 (0·24–0·69)	0·66 (0·39–1·13)
Varenicline	25 561	126	4·9	0·46 (0·38–0·56)	0·73 (0·60–0·88)
**Depression**					
NRT	50 558	8274	163·7	1	1
Bupropion	3162	357	112·9	0·69 (0·62–0·77)	0·75 (0·67–0·83)
Varenicline	24 965	2395	95·9	0·59 (0·56–0·61)	0·66 (0·63–0·69)
**Self-harm**					
NRT	52 832	540	10·2	1	1
Bupropion	3259	20	6·1	0·60 (0·38–0·94)	0·74 (0·48–1·16)
Varenicline	25 570	119	4·7	0·46 (0·37–0·56)	0·56 (0·46–0·68)

Data in parentheses are 95% CIs. NRT=nicotine replacement treatment.

**Table 3:** Hazard ratios of events during 6 months follow-up in the propensity score matched samples

	**Bupropion *vs* NRT (n=12 786)**	**Varenicline *vs* NRT (n=100 326)**
Ischaemic heart disease	0·59 (0·37–0·93)	0·86 (0·76–0·97)
Cerebral infarction	0·46 (0·24–0·89)	0·58 (0·47–0·73)
Heart failure	0·44 (0·14–1·44)	0·64 (0·42–0·98)
Peripheral vascular disease	1·62 (0·67–3·92)	0·95 (0·73–1·23)
Arrhythmia	0·43 (0·21–0·91)	0·72 (0·55–0·92)
Depression	0·80 (0·70–0·92)	0·65 (0·61–0·68)
Self-harm	0·90 (0·49–1·68)	0·60 (0·48–0·76)

Data in parentheses are 95% CIs. NRT=nicotine replacement treatment.

**Table 4:** Hazard ratio for self-harm during 6 months' follow-up in varenicline versus NRT users, adjusted for an unmeasured binary confounder with a hazard ratio of 3^43^

		**P0**										
		0·0	0·1	0·2	0·3	0·4	0·5	0·6	0·7	0·8	0·9	1·0
P1												
	0·0	0·56 (0·46–0·68)	0·67 (0·55–0·82)	0·78 (0·64–0·95)	0·9 (0·74–1·09)	1·01 (0·83–1·22)	1·12 (0·92–1·36)	1·23 (1·01–1·5)*	1·34 (1·1–1·63)*	1·46 (1·2–1·77)*	1·57 (1·29–1·9)*†	1·68 (1·38–2·04)*†
	0·1	0·47 (0·38–0·57)	0·56 (0·46–0·68)	0·65 (0·54–0·79)	0·75 (0·61–0·91)	0·84 (0·69–1·02)	0·93 (0·77–1·13)	1·03 (0·84–1·25)	1·12 (0·92–1·36)	1·21 (1–1·47)*	1·31 (1·07–1·59)*	1·4 (1·15–1·7)*
	0·2	0·4 (0·33–0·49)	0·48 (0·39–0·58)	0·56 (0·46–0·68)	0·64 (0·53–0·78)	0·72 (0·59–0·87)	0·8 (0·66–0·97)	0·88 (0·72–1·07)	0·96 (0·79–1·17)	1·04 (0·85–1·26)	1·12 (0·92–1·36)	1·2 (0·99–1·46)
	0·3	0·35 (0·29–0·43)	0·42 (0·35–0·51)	0·49 (0·4–0·6)	0·56 (0·46–0·68)	0·63 (0·52–0·77)	0·7 (0·58–0·85)	0·77 (0·63–0·94)	0·84 (0·69–1·02)	0·91 (0·75–1·11)	0·98 (0·81–1·19)	1·05 (0·86–1·28)
	0·4	0·31 (0·26–0·38)	0·37 (0·31–0·45)	0·44 (0·36–0·53)	0·5 (0·41–0·6)	0·56 (0·46–0·68)	0·62 (0·51–0·76)	0·68 (0·56–0·83)	0·75 (0·61–0·91)	0·81 (0·66–0·98)	0·87 (0·72–1·06)	0·93 (0·77–1·13)
	0·5	0·28 (0·23–0·34)	0·34 (0·28–0·41)	0·39 (0·32–0·48)	0·45 (0·37–0·54)	0·5 (0·41–0·61)	0·56 (0·46–0·68)	0·62 (0·51–0·75)	0·67 (0·55–0·82)	0·73 (0·6–0·88)	0·78 (0·64–0·95)	0·84 (0·69–1·02)
	0·6	0·25 (0·21–0·31)	0·31 (0·25–0·37)	0·36 (0·29–0·43)	0·41 (0·33–0·49)	0·46 (0·38–0·56)	0·51 (0·42–0·62)	0·56 (0·46–0·68)	0·61 (0·5–0·74)	0·66 (0·54–0·8)	0·71 (0·59–0·87)	0·76 (0·63–0·93)
	0·7	0·23 (0·19–0·28)	0·28 (0·23–0·34)	0·33 (0·27–0·4)	0·37 (0·31–0·45)	0·42 (0·35–0·51)	0·47 (0·38–0·57)	0·51 (0·42–0·62)	0·56 (0·46–0·68)	0·61 (0·5–0·74)	0·65 (0·54–0·79)	0·7 (0·58–0·85)
	0·8	0·22 (0·18–0·26)	0·26 (0·21–0·31)	0·3 (0·25–0·37)	0·34 (0·28–0·42)	0·39 (0·32–0·47)	0·43 (0·35–0·52)	0·47 (0·39–0·58)	0·52 (0·42–0·63)	0·56 (0·46–0·68)	0·6 (0·5–0·73)	0·65 (0·53–0·78)
	0·9	0·2 (0·16–0·24)	0·24 (0·2–0·29)	0·28 (0·23–0·34)	0·32 (0·26–0·39)	0·36 (0·3–0·44)	0·4 (0·33–0·49)	0·44 (0·36–0·53)	0·48 (0·39–0·58)	0·52 (0·43–0·63)	0·56 (0·46–0·68)	0·6 (0·49–0·73)
	1·0	0·19 (0·15–0·23)	0·22 (0·18–0·27)	0·26 (0·21–0·32)	0·3 (0·25–0·36)	0·34 (0·28–0·41)	0·37 (0·31–0·45)	0·41 (0·34–0·5)	0·45 (0·37–0·54)	0·49 (0·4–0·59)	0·52 (0·43–0·63)	0·56 (0·46–0·68)

This table shows how the noted hazard ratio (in the central diagonal line of cells) would change in the presence of an unmeasured confounder with a hazard ratio of 3 and different combinations of prevalences in user groups. P1 and P0 are the prevalences of the unmeasured confounder in varenicline (P1) and NRT (P0) users. Data in parentheses are 95% CIs. NRT=nicotine replacement treatment.

* Varenicline would be associated with a significantly increased hazard of the event.

† The hazard would also be clinically meaningful according to our study protocol (hazard ratio of 1·5 or higher).
