# Supplementary material for: Cardiovascular and neuropsychiatric risks of varenicline: a retrospective cohort study
Source: Lancet Respir Med. 2015 Oct;3(10):761–8. doi: 10.1016/S2213-2600(15)00320-3 (PMC4593936; doi:10.1016/S2213-2600(15)00320-3)
Supplement: Supplementary appendix [file mmc1.pdf]

THE LANCET Respiratory Medicine

Supplementary appendix

This appendix formed part of the original submission and has been peer reviewed. We post it as supplied by the authors.

Supplement to: Kotz D, Viechtbauer W, Simpson C, van Schayck OCP, West R, Sheikh A. Cardiovascular and neuropsychiatric risks of varenicline: a retrospective cohort study. *Lancet Respir Med* 2015; published online Sept 7. http://dx.doi.org/10.1016/S2213-2600(15)00320-3.

Details of the propensity score analyses

Reference to the study protocol: Kotz D, Simpson C, Viechtbauer W, et al. Cardiovascular and neuropsychiatric safety of varenicline and bupropion compared with nicotine replacement therapy for smoking cessation: study protocol of a retrospective cohort study using the QResearch general practice database. BMJ Open 2014; 4

In multiple logistic regression models, medication use (with varenicline vs. NRT Rx as dependent variable in the first model and bupropion vs. NRT Rx in the second model) was regressed on the potential confounders. The resulting predicted probability values for medication use (possible range 0.0 to 1.0) were used as propensity score for using one drug versus the other. In order to estimate how much of the variation in medication use can be explained by the potential confounders, we calculated the area under the receiver operating characteristics (ROC) curve. The possible value from this analysis lies between 0.5 (indicating no association between the propensity score and medication use) and 1.0 (indicating that medication use can be completely explained by the propensity score). We found that the area under the ROC curve was 0.59 for varenicline versus NRT and 0.60 for bupropion versus NRT. We then trimmed the sample by excluding patients with a propensity score corresponding to the 2.5th centile or lower in the varenicline (or bupropion) group, and by excluding patients with a propensity score corresponding to the 97.5th centile or higher in the NRT Rx group. This trimming was intended to exclude patients from the subsequent analyses which used a form of medication strongly contrary to expectation (e.g., a patients who had most of the characteristics associated with the use of varenicline but who used NRT Rx) and may therefore reduce residual confounding. In the propensity score analyses, we then matched patients using varenicline to patients using NRT Rx in a fixed 1:1 ratio by using the nearest neighbour algorithm (MatchIt package in R). Likewise we matched bupropion users with NRT Rx users. In these two matched samples, we again used Cox proportional hazards regression models to assess the association between medication use and each of the above mentioned main outcomes. Hazard ratios were calculated for varenicline and bupropion with NRT Rx as a reference.

Figure E1a: Kaplan-Meier Survival Curves for each Cardiovascular Event During 6 Months Follow-up

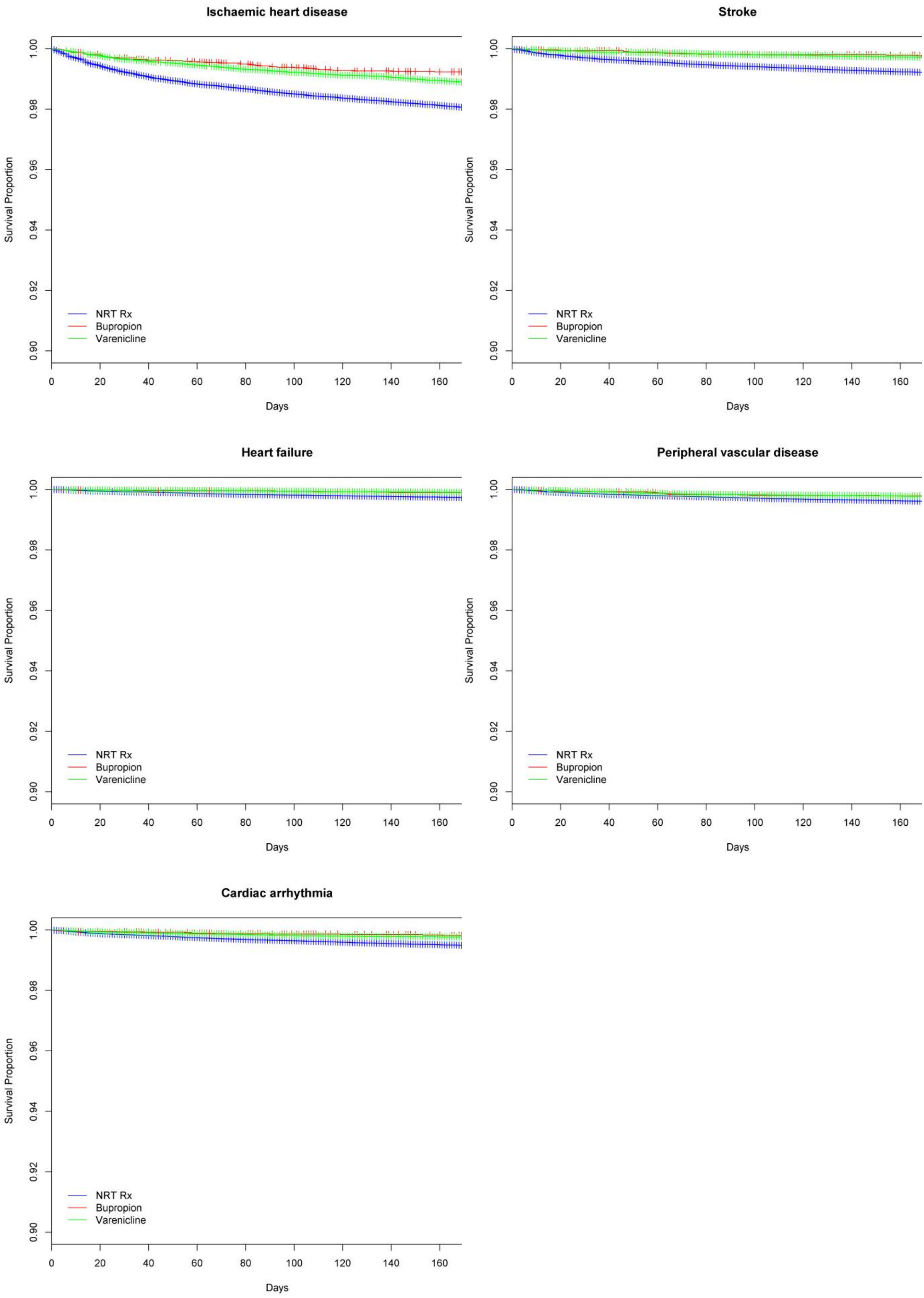

Figure E1b: Kaplan-Meier Survival Curves for each Neuropsychiatric Event During 6 Months Follow-up

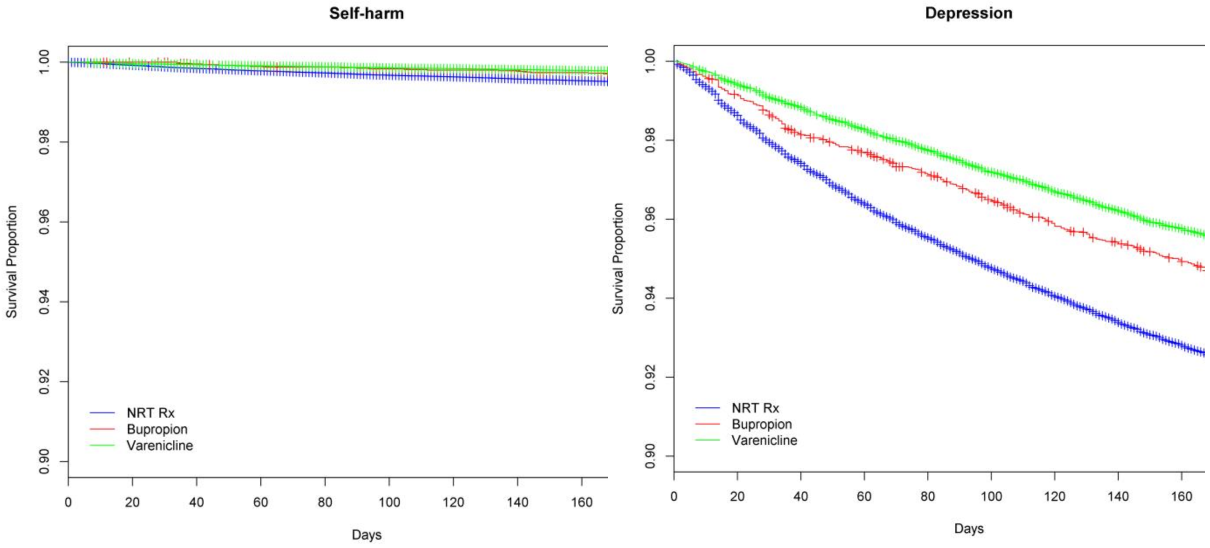

Table E1: Hazard ratio (95%CI) for ischemic heart disease during 6 months follow-up in users of varenicline vs. NRT, adjusted for an unmeasured binary confounder with a hazard ratio of 3

P1 / P0	0.0	0.1	0.2	0.3	0.4	0.5	0.6	0.7	0.8	0.9	1.0
0.0	0.8 (0.72, 0.87)	0.96 (0.86, 1.04)	1.12 (1.01, 1.22)	1.28 (1.15, 1.39)	1.44 (1.3, 1.57)	1.6 (1.44, 1.74)	1.76 (1.58, 1.91)	1.92 (1.73, 2.09)	2.08 (1.87, 2.26)	2.24 (2.02, 2.44)	2.4 (2.16, 2.61)
0.1	0.67 (0.6, 0.73)	0.8 (0.72, 0.87)	0.93 (0.84, 1.02)	1.07 (0.96, 1.16)	1.2 (1.08, 1.31)	1.33 (1.2, 1.45)	1.47 (1.32, 1.6)	1.6 (1.44, 1.74)	1.73 (1.56, 1.89)	1.87 (1.68, 2.03)	2 (1.8, 2.18)
0.2	0.57 (0.51, 0.62)	0.69 (0.62, 0.75)	0.8 (0.72, 0.87)	0.91 (0.82, 0.99)	1.03 (0.93, 1.12)	1.14 (1.03, 1.24)	1.26 (1.13, 1.37)	1.37 (1.23, 1.49)	1.49 (1.34, 1.62)	1.6 (1.44, 1.74)	1.71 (1.54, 1.86)
0.3	0.5 (0.45, 0.54)	0.6 (0.54, 0.65)	0.7 (0.63, 0.76)	0.8 (0.72, 0.87)	0.9 (0.81, 0.98)	1 (0.9, 1.09)	1.1 (0.99, 1.2)	1.2 (1.08, 1.31)	1.3 (1.17, 1.41)	1.4 (1.26, 1.52)	1.5 (1.35, 1.63)
0.4	0.44 (0.4, 0.48)	0.53 (0.48, 0.58)	0.62 (0.56, 0.68)	0.71 (0.64, 0.77)	0.8 (0.72, 0.87)	0.89 (0.8, 0.97)	0.98 (0.88, 1.06)	1.07 (0.96, 1.16)	1.16 (1.04, 1.26)	1.24 (1.12, 1.35)	1.33 (1.2, 1.45)
0.5	0.4 (0.36, 0.44)	0.48 (0.43, 0.52)	0.56 (0.5, 0.61)	0.64 (0.58, 0.7)	0.72 (0.65, 0.78)	0.8 (0.72, 0.87)	0.88 (0.79, 0.96)	0.96 (0.86, 1.04)	1.04 (0.94, 1.13)	1.12 (1.01, 1.22)	1.2 (1.08, 1.31)
0.6	0.36 (0.33, 0.4)	0.44 (0.39, 0.47)	0.51 (0.46, 0.55)	0.58 (0.52, 0.63)	0.65 (0.59, 0.71)	0.73 (0.65, 0.79)	0.8 (0.72, 0.87)	0.87 (0.79, 0.95)	0.95 (0.85, 1.03)	1.02 (0.92, 1.11)	1.09 (0.98, 1.19)
0.7	0.33 (0.3, 0.36)	0.4 (0.36, 0.44)	0.47 (0.42, 0.51)	0.53 (0.48, 0.58)	0.6 (0.54, 0.65)	0.67 (0.6, 0.73)	0.73 (0.66, 0.8)	0.8 (0.72, 0.87)	0.87 (0.78, 0.94)	0.93 (0.84, 1.02)	1 (0.9, 1.09)
0.8	0.31 (0.28, 0.33)	0.37 (0.33, 0.4)	0.43 (0.39, 0.47)	0.49 (0.44, 0.54)	0.55 (0.5, 0.6)	0.62 (0.55, 0.67)	0.68 (0.61, 0.74)	0.74 (0.66, 0.8)	0.8 (0.72, 0.87)	0.86 (0.78, 0.94)	0.92 (0.83, 1)
0.9	0.29 (0.26, 0.31)	0.34 (0.31, 0.37)	0.4 (0.36, 0.44)	0.46 (0.41, 0.5)	0.51 (0.46, 0.56)	0.57 (0.51, 0.62)	0.63 (0.57, 0.68)	0.69 (0.62, 0.75)	0.74 (0.67, 0.81)	0.8 (0.72, 0.87)	0.86 (0.77, 0.93)
1.0	0.27 (0.24, 0.29)	0.32 (0.29, 0.35)	0.37 (0.34, 0.41)	0.43 (0.38, 0.46)	0.48 (0.43, 0.52)	0.53 (0.48, 0.58)	0.59 (0.53, 0.64)	0.64 (0.58, 0.7)	0.69 (0.62, 0.75)	0.75 (0.67, 0.81)	0.8 (0.72, 0.87)

This tables shows how the observed hazard ratio (diagonal line of cells filled with light blue) would change in the presence of an unmeasured confounder with a hazard ratio of 3 and different combinations of prevalence rates among the user groups. P1/P0 = prevalence of the unmeasured confounder among users of varenicline (P1) and NRT (P0). The cells filled with red mark the situations in which varenicline would be associated with a statistically significant increased hazard of the event. In addition, the cells filled with dark red mark the situations in which this hazard would also be clinically meaningful according to our study protocol (HR>=1.5). These calculations are based on: Lin et al. Biometrics 1998, 54(3), 948-963 (equation 2.9).

Table E2: Hazard ratio (95%CI) for cerebral infarction during 6 months follow-up in users of varenicline vs. NRT, adjusted for an unmeasured binary confounder with a hazard ratio of 3

P1 / P0	0.0	0.1	0.2	0.3	0.4	0.5	0.6	0.7	0.8	0.9	1.0
0.0	0.62 (0.52, 0.73)	0.74 (0.62, 0.88)	0.87 (0.73, 1.02)	0.99 (0.83, 1.17)	1.12 (0.94, 1.31)	1.24 (1.04, 1.46)	1.36 (1.14, 1.61)	1.49 (1.25, 1.75)	1.61 (1.35, 1.9)	1.74 (1.46, 2.04)	1.86 (1.56, 2.19)
0.1	0.52 (0.43, 0.61)	0.62 (0.52, 0.73)	0.72 (0.61, 0.85)	0.83 (0.69, 0.97)	0.93 (0.78, 1.1)	1.03 (0.87, 1.22)	1.14 (0.95, 1.34)	1.24 (1.04, 1.46)	1.34 (1.13, 1.58)	1.45 (1.21, 1.7)	1.55 (1.3, 1.83)
0.2	0.44 (0.37, 0.52)	0.53 (0.45, 0.63)	0.62 (0.52, 0.73)	0.71 (0.59, 0.83)	0.8 (0.67, 0.94)	0.89 (0.74, 1.04)	0.97 (0.82, 1.15)	1.06 (0.89, 1.25)	1.15 (0.97, 1.36)	1.24 (1.04, 1.46)	1.33 (1.11, 1.56)
0.3	0.39 (0.33, 0.46)	0.47 (0.39, 0.55)	0.54 (0.46, 0.64)	0.62 (0.52, 0.73)	0.7 (0.59, 0.82)	0.78 (0.65, 0.91)	0.85 (0.72, 1)	0.93 (0.78, 1.1)	1.01 (0.85, 1.19)	1.09 (0.91, 1.28)	1.16 (0.98, 1.37)
0.4	0.34 (0.29, 0.41)	0.41 (0.35, 0.49)	0.48 (0.4, 0.57)	0.55 (0.46, 0.65)	0.62 (0.52, 0.73)	0.69 (0.58, 0.81)	0.76 (0.64, 0.89)	0.83 (0.69, 0.97)	0.9 (0.75, 1.05)	0.96 (0.81, 1.14)	1.03 (0.87, 1.22)
0.5	0.31 (0.26, 0.37)	0.37 (0.31, 0.44)	0.43 (0.36, 0.51)	0.5 (0.42, 0.58)	0.56 (0.47, 0.66)	0.62 (0.52, 0.73)	0.68 (0.57, 0.8)	0.74 (0.62, 0.88)	0.81 (0.68, 0.95)	0.87 (0.73, 1.02)	0.93 (0.78, 1.1)
0.6	0.28 (0.24, 0.33)	0.34 (0.28, 0.4)	0.39 (0.33, 0.46)	0.45 (0.38, 0.53)	0.51 (0.43, 0.6)	0.56 (0.47, 0.66)	0.62 (0.52, 0.73)	0.68 (0.57, 0.8)	0.73 (0.61, 0.86)	0.79 (0.66, 0.93)	0.85 (0.71, 1)
0.7	0.26 (0.22, 0.3)	0.31 (0.26, 0.37)	0.36 (0.3, 0.43)	0.41 (0.35, 0.49)	0.47 (0.39, 0.55)	0.52 (0.43, 0.61)	0.57 (0.48, 0.67)	0.62 (0.52, 0.73)	0.67 (0.56, 0.79)	0.72 (0.61, 0.85)	0.78 (0.65, 0.91)
0.8	0.24 (0.2, 0.28)	0.29 (0.24, 0.34)	0.33 (0.28, 0.39)	0.38 (0.32, 0.45)	0.43 (0.36, 0.51)	0.48 (0.4, 0.56)	0.52 (0.44, 0.62)	0.57 (0.48, 0.67)	0.62 (0.52, 0.73)	0.67 (0.56, 0.79)	0.72 (0.6, 0.84)
0.9	0.22 (0.19, 0.26)	0.27 (0.22, 0.31)	0.31 (0.26, 0.37)	0.35 (0.3, 0.42)	0.4 (0.33, 0.47)	0.44 (0.37, 0.52)	0.49 (0.41, 0.57)	0.53 (0.45, 0.63)	0.58 (0.48, 0.68)	0.62 (0.52, 0.73)	0.66 (0.56, 0.78)
1.0	0.21 (0.17, 0.24)	0.25 (0.21, 0.29)	0.29 (0.24, 0.34)	0.33 (0.28, 0.39)	0.37 (0.31, 0.44)	0.41 (0.35, 0.49)	0.45 (0.38, 0.54)	0.5 (0.42, 0.58)	0.54 (0.45, 0.63)	0.58 (0.49, 0.68)	0.62 (0.52, 0.73)

This tables shows how the observed hazard ratio (diagonal line of cells filled with light blue) would change in the presence of an unmeasured confounder with a hazard ratio of 3 and different combinations of prevalence rates among the user groups. P1/P0 = prevalence of the unmeasured confounder among users of varenicline (P1) and NRT (P0). The cells filled with red mark the situations in which varenicline would be associated with a statistically significant increased hazard of the event. In addition, the cells filled with dark red mark the situations in which this hazard would also be clinically meaningful according to our study protocol (HR>=1.5). These calculations are based on: Lin et al. Biometrics 1998, 54(3), 948-963 (equation 2.9).

Table E3: Hazard ratio (95%CI) for heart failure during 6 months follow-up in users of varenicline vs. NRT, adjusted for an unmeasured binary confounder with a hazard ratio of 3

P1 / P0	0.0	0.1	0.2	0.3	0.4	0.5	0.6	0.7	0.8	0.9	1.0
0.0	0.61 (0.45, 0.83)	0.73 (0.54, 1)	0.85 (0.63, 1.16)	0.98 (0.72, 1.33)	1.1 (0.81, 1.49)	1.22 (0.9, 1.66)	1.34 (0.99, 1.83)	1.46 (1.08, 1.99)	1.59 (1.17, 2.16)	1.71 (1.26, 2.32)	1.83 (1.35, 2.49)
0.1	0.51 (0.38, 0.69)	0.61 (0.45, 0.83)	0.71 (0.53, 0.97)	0.81 (0.6, 1.11)	0.92 (0.68, 1.25)	1.02 (0.75, 1.38)	1.12 (0.83, 1.52)	1.22 (0.9, 1.66)	1.32 (0.98, 1.8)	1.42 (1.05, 1.94)	1.53 (1.13, 2.08)
0.2	0.44 (0.32, 0.59)	0.52 (0.39, 0.71)	0.61 (0.45, 0.83)	0.7 (0.51, 0.95)	0.78 (0.58, 1.07)	0.87 (0.64, 1.19)	0.96 (0.71, 1.3)	1.05 (0.77, 1.42)	1.13 (0.84, 1.54)	1.22 (0.9, 1.66)	1.31 (0.96, 1.78)
0.3	0.38 (0.28, 0.52)	0.46 (0.34, 0.62)	0.53 (0.39, 0.73)	0.61 (0.45, 0.83)	0.69 (0.51, 0.93)	0.76 (0.56, 1.04)	0.84 (0.62, 1.14)	0.92 (0.68, 1.25)	0.99 (0.73, 1.35)	1.07 (0.79, 1.45)	1.14 (0.84, 1.56)
0.4	0.34 (0.25, 0.46)	0.41 (0.3, 0.55)	0.47 (0.35, 0.65)	0.54 (0.4, 0.74)	0.61 (0.45, 0.83)	0.68 (0.5, 0.92)	0.75 (0.55, 1.01)	0.81 (0.6, 1.11)	0.88 (0.65, 1.2)	0.95 (0.7, 1.29)	1.02 (0.75, 1.38)
0.5	0.31 (0.23, 0.42)	0.37 (0.27, 0.5)	0.43 (0.32, 0.58)	0.49 (0.36, 0.66)	0.55 (0.41, 0.75)	0.61 (0.45, 0.83)	0.67 (0.5, 0.91)	0.73 (0.54, 1)	0.79 (0.59, 1.08)	0.85 (0.63, 1.16)	0.92 (0.68, 1.25)
0.6	0.28 (0.2, 0.38)	0.33 (0.25, 0.45)	0.39 (0.29, 0.53)	0.44 (0.33, 0.6)	0.5 (0.37, 0.68)	0.55 (0.41, 0.75)	0.61 (0.45, 0.83)	0.67 (0.49, 0.91)	0.72 (0.53, 0.98)	0.78 (0.57, 1.06)	0.83 (0.61, 1.13)
0.7	0.25 (0.19, 0.35)	0.31 (0.23, 0.42)	0.36 (0.26, 0.48)	0.41 (0.3, 0.55)	0.46 (0.34, 0.62)	0.51 (0.38, 0.69)	0.56 (0.41, 0.76)	0.61 (0.45, 0.83)	0.66 (0.49, 0.9)	0.71 (0.53, 0.97)	0.76 (0.56, 1.04)
0.8	0.23 (0.17, 0.32)	0.28 (0.21, 0.38)	0.33 (0.24, 0.45)	0.38 (0.28, 0.51)	0.42 (0.31, 0.57)	0.47 (0.35, 0.64)	0.52 (0.38, 0.7)	0.56 (0.42, 0.77)	0.61 (0.45, 0.83)	0.66 (0.48, 0.89)	0.7 (0.52, 0.96)
0.9	0.22 (0.16, 0.3)	0.26 (0.19, 0.36)	0.31 (0.23, 0.42)	0.35 (0.26, 0.47)	0.39 (0.29, 0.53)	0.44 (0.32, 0.59)	0.48 (0.35, 0.65)	0.52 (0.39, 0.71)	0.57 (0.42, 0.77)	0.61 (0.45, 0.83)	0.65 (0.48, 0.89)
1.0	0.2 (0.15, 0.28)	0.24 (0.18, 0.33)	0.28 (0.21, 0.39)	0.33 (0.24, 0.44)	0.37 (0.27, 0.5)	0.41 (0.3, 0.55)	0.45 (0.33, 0.61)	0.49 (0.36, 0.66)	0.53 (0.39, 0.72)	0.57 (0.42, 0.77)	0.61 (0.45, 0.83)

This tables shows how the observed hazard ratio (diagonal line of cells filled with light blue) would change in the presence of an unmeasured confounder with a hazard ratio of 3 and different combinations of prevalence rates among the user groups. P1/P0 = prevalence of the unmeasured confounder among users of varenicline (P1) and NRT (P0). The cells filled with red mark the situations in which varenicline would be associated with a statistically significant increased hazard of the event. In addition, the cells filled with dark red mark the situations in which this hazard would also be clinically meaningful according to our study protocol (HR>=1.5). These calculations are based on: Lin et al. Biometrics 1998, 54(3), 948-963 (equation 2.9).

Table E4: Hazard ratio (95%CI) for peripheral vascular disease during 6 months follow-up in users of varenicline vs. NRT, adjusted for an unmeasured binary confounder with a hazard ratio of 3

P1 / P0	0.0	0.1	0.2	0.3	0.4	0.5	0.6	0.7	0.8	0.9	1.0
0.0	0.82 (0.67, 1.01)	0.98 (0.8, 1.21)	1.15 (0.94, 1.41)	1.31 (1.07, 1.62)	1.48 (1.21, 1.82)	1.64 (1.34, 2.02)	1.8 (1.47, 2.22)	1.97 (1.61, 2.42)	2.13 (1.74, 2.63)	2.3 (1.88, 2.83)	2.46 (2.01, 3.03)
0.1	0.68 (0.56, 0.84)	0.82 (0.67, 1.01)	0.96 (0.78, 1.18)	1.09 (0.89, 1.35)	1.23 (1.01, 1.52)	1.37 (1.12, 1.68)	1.5 (1.23, 1.85)	1.64 (1.34, 2.02)	1.78 (1.45, 2.19)	1.91 (1.56, 2.36)	2.05 (1.68, 2.53)
0.2	0.59 (0.48, 0.72)	0.7 (0.57, 0.87)	0.82 (0.67, 1.01)	0.94 (0.77, 1.15)	1.05 (0.86, 1.3)	1.17 (0.96, 1.44)	1.29 (1.05, 1.59)	1.41 (1.15, 1.73)	1.52 (1.24, 1.88)	1.64 (1.34, 2.02)	1.76 (1.44, 2.16)
0.3	0.51 (0.42, 0.63)	0.62 (0.5, 0.76)	0.72 (0.59, 0.88)	0.82 (0.67, 1.01)	0.92 (0.75, 1.14)	1.03 (0.84, 1.26)	1.13 (0.92, 1.39)	1.23 (1.01, 1.52)	1.33 (1.09, 1.64)	1.44 (1.17, 1.77)	1.54 (1.26, 1.89)
0.4	0.46 (0.37, 0.56)	0.55 (0.45, 0.67)	0.64 (0.52, 0.79)	0.73 (0.6, 0.9)	0.82 (0.67, 1.01)	0.91 (0.74, 1.12)	1 (0.82, 1.23)	1.09 (0.89, 1.35)	1.18 (0.97, 1.46)	1.28 (1.04, 1.57)	1.37 (1.12, 1.68)
0.5	0.41 (0.34, 0.51)	0.49 (0.4, 0.61)	0.57 (0.47, 0.71)	0.66 (0.54, 0.81)	0.74 (0.6, 0.91)	0.82 (0.67, 1.01)	0.9 (0.74, 1.11)	0.98 (0.8, 1.21)	1.07 (0.87, 1.31)	1.15 (0.94, 1.41)	1.23 (1.01, 1.52)
0.6	0.37 (0.3, 0.46)	0.45 (0.37, 0.55)	0.52 (0.43, 0.64)	0.6 (0.49, 0.73)	0.67 (0.55, 0.83)	0.75 (0.61, 0.92)	0.82 (0.67, 1.01)	0.89 (0.73, 1.1)	0.97 (0.79, 1.19)	1.04 (0.85, 1.29)	1.12 (0.91, 1.38)
0.7	0.34 (0.28, 0.42)	0.41 (0.34, 0.51)	0.48 (0.39, 0.59)	0.55 (0.45, 0.67)	0.62 (0.5, 0.76)	0.68 (0.56, 0.84)	0.75 (0.61, 0.93)	0.82 (0.67, 1.01)	0.89 (0.73, 1.09)	0.96 (0.78, 1.18)	1.03 (0.84, 1.26)
0.8	0.32 (0.26, 0.39)	0.38 (0.31, 0.47)	0.44 (0.36, 0.54)	0.5 (0.41, 0.62)	0.57 (0.46, 0.7)	0.63 (0.52, 0.78)	0.69 (0.57, 0.85)	0.76 (0.62, 0.93)	0.82 (0.67, 1.01)	0.88 (0.72, 1.09)	0.95 (0.77, 1.17)
0.9	0.29 (0.24, 0.36)	0.35 (0.29, 0.43)	0.41 (0.34, 0.51)	0.47 (0.38, 0.58)	0.53 (0.43, 0.65)	0.59 (0.48, 0.72)	0.64 (0.53, 0.79)	0.7 (0.57, 0.87)	0.76 (0.62, 0.94)	0.82 (0.67, 1.01)	0.88 (0.72, 1.08)
1.0	0.27 (0.22, 0.34)	0.33 (0.27, 0.4)	0.38 (0.31, 0.47)	0.44 (0.36, 0.54)	0.49 (0.4, 0.61)	0.55 (0.45, 0.67)	0.6 (0.49, 0.74)	0.66 (0.54, 0.81)	0.71 (0.58, 0.88)	0.77 (0.63, 0.94)	0.82 (0.67, 1.01)

This tables shows how the observed hazard ratio (diagonal line of cells filled with light blue) would change in the presence of an unmeasured confounder with a hazard ratio of 3 and different combinations of prevalence rates among the user groups. P1/P0 = prevalence of the unmeasured confounder among users of varenicline (P1) and NRT (P0). The cells filled with red mark the situations in which varenicline would be associated with a statistically significant increased hazard of the event. In addition, the cells filled with dark red mark the situations in which this hazard would also be clinically meaningful according to our study protocol (HR>=1.5). These calculations are based on: Lin et al. Biometrics 1998, 54(3), 948-963 (equation 2.9).

Table E5: Hazard ratio (95%CI) for arrhythmia during 6 months follow-up in users of varenicline vs. NRT, adjusted for an unmeasured binary confounder with a hazard ratio of 3

P1 / P0	0.0	0.1	0.2	0.3	0.4	0.5	0.6	0.7	0.8	0.9	1.0
0.0	0.73 (0.6, 0.88)	0.88 (0.72, 1.06)	1.02 (0.84, 1.23)	1.17 (0.96, 1.41)	1.31 (1.08, 1.58)	1.46 (1.2, 1.76)	1.61 (1.32, 1.94)	1.75 (1.44, 2.11)	1.9 (1.56, 2.29)	2.04 (1.68, 2.46)	2.19 (1.8, 2.64)
0.1	0.61 (0.5, 0.73)	0.73 (0.6, 0.88)	0.85 (0.7, 1.03)	0.97 (0.8, 1.17)	1.1 (0.9, 1.32)	1.22 (1, 1.47)	1.34 (1.1, 1.61)	1.46 (1.2, 1.76)	1.58 (1.3, 1.91)	1.7 (1.4, 2.05)	1.83 (1.5, 2.2)
0.2	0.52 (0.43, 0.63)	0.63 (0.51, 0.75)	0.73 (0.6, 0.88)	0.83 (0.69, 1.01)	0.94 (0.77, 1.13)	1.04 (0.86, 1.26)	1.15 (0.94, 1.38)	1.25 (1.03, 1.51)	1.36 (1.11, 1.63)	1.46 (1.2, 1.76)	1.56 (1.29, 1.89)
0.3	0.46 (0.38, 0.55)	0.55 (0.45, 0.66)	0.64 (0.53, 0.77)	0.73 (0.6, 0.88)	0.82 (0.68, 0.99)	0.91 (0.75, 1.1)	1 (0.83, 1.21)	1.1 (0.9, 1.32)	1.19 (0.98, 1.43)	1.28 (1.05, 1.54)	1.37 (1.13, 1.65)
0.4	0.41 (0.33, 0.49)	0.49 (0.4, 0.59)	0.57 (0.47, 0.68)	0.65 (0.53, 0.78)	0.73 (0.6, 0.88)	0.81 (0.67, 0.98)	0.89 (0.73, 1.08)	0.97 (0.8, 1.17)	1.05 (0.87, 1.27)	1.14 (0.93, 1.37)	1.22 (1, 1.47)
0.5	0.37 (0.3, 0.44)	0.44 (0.36, 0.53)	0.51 (0.42, 0.62)	0.58 (0.48, 0.7)	0.66 (0.54, 0.79)	0.73 (0.6, 0.88)	0.8 (0.66, 0.97)	0.88 (0.72, 1.06)	0.95 (0.78, 1.14)	1.02 (0.84, 1.23)	1.1 (0.9, 1.32)
0.6	0.33 (0.27, 0.4)	0.4 (0.33, 0.48)	0.46 (0.38, 0.56)	0.53 (0.44, 0.64)	0.6 (0.49, 0.72)	0.66 (0.55, 0.8)	0.73 (0.6, 0.88)	0.8 (0.65, 0.96)	0.86 (0.71, 1.04)	0.93 (0.76, 1.12)	1 (0.82, 1.2)
0.7	0.3 (0.25, 0.37)	0.37 (0.3, 0.44)	0.43 (0.35, 0.51)	0.49 (0.4, 0.59)	0.55 (0.45, 0.66)	0.61 (0.5, 0.73)	0.67 (0.55, 0.81)	0.73 (0.6, 0.88)	0.79 (0.65, 0.95)	0.85 (0.7, 1.03)	0.91 (0.75, 1.1)
0.8	0.28 (0.23, 0.34)	0.34 (0.28, 0.41)	0.39 (0.32, 0.47)	0.45 (0.37, 0.54)	0.51 (0.42, 0.61)	0.56 (0.46, 0.68)	0.62 (0.51, 0.74)	0.67 (0.55, 0.81)	0.73 (0.6, 0.88)	0.79 (0.65, 0.95)	0.84 (0.69, 1.02)
0.9	0.26 (0.21, 0.31)	0.31 (0.26, 0.38)	0.37 (0.3, 0.44)	0.42 (0.34, 0.5)	0.47 (0.39, 0.57)	0.52 (0.43, 0.63)	0.57 (0.47, 0.69)	0.63 (0.51, 0.75)	0.68 (0.56, 0.82)	0.73 (0.6, 0.88)	0.78 (0.64, 0.94)
1.0	0.24 (0.2, 0.29)	0.29 (0.24, 0.35)	0.34 (0.28, 0.41)	0.39 (0.32, 0.47)	0.44 (0.36, 0.53)	0.49 (0.4, 0.59)	0.54 (0.44, 0.65)	0.58 (0.48, 0.7)	0.63 (0.52, 0.76)	0.68 (0.56, 0.82)	0.73 (0.6, 0.88)

This tables shows how the observed hazard ratio (diagonal line of cells filled with light blue) would change in the presence of an unmeasured confounder with a hazard ratio of 3 and different combinations of prevalence rates among the user groups. P1/P0 = prevalence of the unmeasured confounder among users of varenicline (P1) and NRT (P0). The cells filled with red mark the situations in which varenicline would be associated with a statistically significant increased hazard of the event. In addition, the cells filled with dark red mark the situations in which this hazard would also be clinically meaningful according to our study protocol (HR>=1.5). These calculations are based on: Lin et al. Biometrics 1998, 54(3), 948-963 (equation 2.9).

Table E6: Hazard ratio (95%CI) for depression during 6 months follow-up in users of varenicline vs. NRT, adjusted for an unmeasured binary confounder with a hazard ratio of 3

P1 / P0	0.0	0.1	0.2	0.3	0.4	0.5	0.6	0.7	0.8	0.9	1.0
0.0	0.66 (0.63, 0.69)	0.79 (0.76, 0.83)	0.92 (0.88, 0.97)	1.06 (1.01, 1.1)	1.19 (1.13, 1.24)	1.32 (1.26, 1.38)	1.45 (1.39, 1.52)	1.58 (1.51, 1.66)	1.72 (1.64, 1.79)	1.85 (1.76, 1.93)	1.98 (1.89, 2.07)
0.1	0.55 (0.53, 0.58)	0.66 (0.63, 0.69)	0.77 (0.74, 0.81)	0.88 (0.84, 0.92)	0.99 (0.95, 1.04)	1.1 (1.05, 1.15)	1.21 (1.16, 1.27)	1.32 (1.26, 1.38)	1.43 (1.37, 1.5)	1.54 (1.47, 1.61)	1.65 (1.58, 1.73)
0.2	0.47 (0.45, 0.49)	0.57 (0.54, 0.59)	0.66 (0.63, 0.69)	0.75 (0.72, 0.79)	0.85 (0.81, 0.89)	0.94 (0.9, 0.99)	1.04 (0.99, 1.08)	1.13 (1.08, 1.18)	1.23 (1.17, 1.28)	1.32 (1.26, 1.38)	1.41 (1.35, 1.48)
0.3	0.41 (0.39, 0.43)	0.5 (0.47, 0.52)	0.58 (0.55, 0.6)	0.66 (0.63, 0.69)	0.74 (0.71, 0.78)	0.83 (0.79, 0.86)	0.91 (0.87, 0.95)	0.99 (0.95, 1.04)	1.07 (1.02, 1.12)	1.16 (1.1, 1.21)	1.24 (1.18, 1.29)
0.4	0.37 (0.35, 0.38)	0.44 (0.42, 0.46)	0.51 (0.49, 0.54)	0.59 (0.56, 0.61)	0.66 (0.63, 0.69)	0.73 (0.7, 0.77)	0.81 (0.77, 0.84)	0.88 (0.84, 0.92)	0.95 (0.91, 1)	1.03 (0.98, 1.07)	1.1 (1.05, 1.15)
0.5	0.33 (0.32, 0.35)	0.4 (0.38, 0.41)	0.46 (0.44, 0.48)	0.53 (0.5, 0.55)	0.59 (0.57, 0.62)	0.66 (0.63, 0.69)	0.73 (0.69, 0.76)	0.79 (0.76, 0.83)	0.86 (0.82, 0.9)	0.92 (0.88, 0.97)	0.99 (0.95, 1.04)
0.6	0.3 (0.29, 0.31)	0.36 (0.34, 0.38)	0.42 (0.4, 0.44)	0.48 (0.46, 0.5)	0.54 (0.52, 0.56)	0.6 (0.57, 0.63)	0.66 (0.63, 0.69)	0.72 (0.69, 0.75)	0.78 (0.74, 0.82)	0.84 (0.8, 0.88)	0.9 (0.86, 0.94)
0.7	0.28 (0.26, 0.29)	0.33 (0.32, 0.35)	0.39 (0.37, 0.4)	0.44 (0.42, 0.46)	0.5 (0.47, 0.52)	0.55 (0.53, 0.58)	0.61 (0.58, 0.63)	0.66 (0.63, 0.69)	0.72 (0.68, 0.75)	0.77 (0.74, 0.81)	0.83 (0.79, 0.86)
0.8	0.25 (0.24, 0.27)	0.3 (0.29, 0.32)	0.36 (0.34, 0.37)	0.41 (0.39, 0.42)	0.46 (0.44, 0.48)	0.51 (0.48, 0.53)	0.56 (0.53, 0.58)	0.61 (0.58, 0.64)	0.66 (0.63, 0.69)	0.71 (0.68, 0.74)	0.76 (0.73, 0.8)
0.9	0.24 (0.23, 0.25)	0.28 (0.27, 0.3)	0.33 (0.32, 0.35)	0.38 (0.36, 0.39)	0.42 (0.41, 0.44)	0.47 (0.45, 0.49)	0.52 (0.5, 0.54)	0.57 (0.54, 0.59)	0.61 (0.59, 0.64)	0.66 (0.63, 0.69)	0.71 (0.68, 0.74)
1.0	0.22 (0.21, 0.23)	0.26 (0.25, 0.28)	0.31 (0.29, 0.32)	0.35 (0.34, 0.37)	0.4 (0.38, 0.41)	0.44 (0.42, 0.46)	0.48 (0.46, 0.51)	0.53 (0.5, 0.55)	0.57 (0.55, 0.6)	0.62 (0.59, 0.64)	0.66 (0.63, 0.69)

This tables shows how the observed hazard ratio (diagonal line of cells filled with light blue) would change in the presence of an unmeasured confounder with a hazard ratio of 3 and different combinations of prevalence rates among the user groups. P1/P0 = prevalence of the unmeasured confounder among users of varenicline (P1) and NRT (P0). The cells filled with red mark the situations in which varenicline would be associated with a statistically significant increased hazard of the event. In addition, the cells filled with dark red mark the situations in which this hazard would also be clinically meaningful according to our study protocol (HR>=1.5). These calculations are based on: Lin et al. Biometrics 1998, 54(3), 948-963 (equation 2.9).

Table E7: Hazard ratio (95%CI) for self-harm during 6 months follow-up in users of varenicline vs. NRT, adjusted for an unmeasured binary confounder with a hazard ratio of 3

P1 / P0	0.0	0.1	0.2	0.3	0.4	0.5	0.6	0.7	0.8	0.9	1.0
0.0	0.56 (0.46, 0.68)	0.67 (0.55, 0.82)	0.78 (0.64, 0.95)	0.9 (0.74, 1.09)	1.01 (0.83, 1.22)	1.12 (0.92, 1.36)	1.23 (1.01, 1.5)	1.34 (1.1, 1.63)	1.46 (1.2, 1.77)	1.57 (1.29, 1.9)	1.68 (1.38, 2.04)
0.1	0.47 (0.38, 0.57)	0.56 (0.46, 0.68)	0.65 (0.54, 0.79)	0.75 (0.61, 0.91)	0.84 (0.69, 1.02)	0.93 (0.77, 1.13)	1.03 (0.84, 1.25)	1.12 (0.92, 1.36)	1.21 (1, 1.47)	1.31 (1.07, 1.59)	1.4 (1.15, 1.7)
0.2	0.4 (0.33, 0.49)	0.48 (0.39, 0.58)	0.56 (0.46, 0.68)	0.64 (0.53, 0.78)	0.72 (0.59, 0.87)	0.8 (0.66, 0.97)	0.88 (0.72, 1.07)	0.96 (0.79, 1.17)	1.04 (0.85, 1.26)	1.12 (0.92, 1.36)	1.2 (0.99, 1.46)
0.3	0.35 (0.29, 0.43)	0.42 (0.35, 0.51)	0.49 (0.4, 0.6)	0.56 (0.46, 0.68)	0.63 (0.52, 0.77)	0.7 (0.58, 0.85)	0.77 (0.63, 0.94)	0.84 (0.69, 1.02)	0.91 (0.75, 1.11)	0.98 (0.81, 1.19)	1.05 (0.86, 1.28)
0.4	0.31 (0.26, 0.38)	0.37 (0.31, 0.45)	0.44 (0.36, 0.53)	0.5 (0.41, 0.6)	0.56 (0.46, 0.68)	0.62 (0.51, 0.76)	0.68 (0.56, 0.83)	0.75 (0.61, 0.91)	0.81 (0.66, 0.98)	0.87 (0.72, 1.06)	0.93 (0.77, 1.13)
0.5	0.28 (0.23, 0.34)	0.34 (0.28, 0.41)	0.39 (0.32, 0.48)	0.45 (0.37, 0.54)	0.5 (0.41, 0.61)	0.56 (0.46, 0.68)	0.62 (0.51, 0.75)	0.67 (0.55, 0.82)	0.73 (0.6, 0.88)	0.78 (0.64, 0.95)	0.84 (0.69, 1.02)
0.6	0.25 (0.21, 0.31)	0.31 (0.25, 0.37)	0.36 (0.29, 0.43)	0.41 (0.33, 0.49)	0.46 (0.38, 0.56)	0.51 (0.42, 0.62)	0.56 (0.46, 0.68)	0.61 (0.5, 0.74)	0.66 (0.54, 0.8)	0.71 (0.59, 0.87)	0.76 (0.63, 0.93)
0.7	0.23 (0.19, 0.28)	0.28 (0.23, 0.34)	0.33 (0.27, 0.4)	0.37 (0.31, 0.45)	0.42 (0.35, 0.51)	0.47 (0.38, 0.57)	0.51 (0.42, 0.62)	0.56 (0.46, 0.68)	0.61 (0.5, 0.74)	0.65 (0.54, 0.79)	0.7 (0.58, 0.85)
0.8	0.22 (0.18, 0.26)	0.26 (0.21, 0.31)	0.3 (0.25, 0.37)	0.34 (0.28, 0.42)	0.39 (0.32, 0.47)	0.43 (0.35, 0.52)	0.47 (0.39, 0.58)	0.52 (0.42, 0.63)	0.56 (0.46, 0.68)	0.6 (0.5, 0.73)	0.65 (0.53, 0.78)
0.9	0.2 (0.16, 0.24)	0.24 (0.2, 0.29)	0.28 (0.23, 0.34)	0.32 (0.26, 0.39)	0.36 (0.3, 0.44)	0.4 (0.33, 0.49)	0.44 (0.36, 0.53)	0.48 (0.39, 0.58)	0.52 (0.43, 0.63)	0.56 (0.46, 0.68)	0.6 (0.49, 0.73)
1.0	0.19 (0.15, 0.23)	0.22 (0.18, 0.27)	0.26 (0.21, 0.32)	0.3 (0.25, 0.36)	0.34 (0.28, 0.41)	0.37 (0.31, 0.45)	0.41 (0.34, 0.5)	0.45 (0.37, 0.54)	0.49 (0.4, 0.59)	0.52 (0.43, 0.63)	0.56 (0.46, 0.68)

This tables shows how the observed hazard ratio (diagonal line of cells filled with light blue) would change in the presence of an unmeasured confounder with a hazard ratio of 3 and different combinations of prevalence rates among the user groups. P1/P0 = prevalence of the unmeasured confounder among users of varenicline (P1) and NRT Rx (P0). The cells filled with red mark the situations in which varenicline would be associated with a statistically significant increased hazard of the event. In addition, the cells filled with dark red mark the situations in which this hazard would also be clinically meaningful according to our study protocol (HR>=1.5). These calculations are based on: Lin et al. Biometrics 1998, 54(3), 948-963 (equation 2.9).

Table E8: Incidence Rates of Events and Hazard Ratios (95%CI) of Medication Groups for all Events During 3 Months Follow-up

Event	Patient-years	Number of events	Incidence of event per 1,000 patient-years	Hazard ratio (95% CI)	
				Crude	Adjusted*
Ischemic heart disease					
NRT	26,242	1,516	57.8	1	1
Bupropion	1,624	38	23.4	0.41 (0.29-0.56)	0.69 (0.50-0.95)
Varenicline	12,713	372	29.3	0.51 (0.45-0.57)	0.69 (0.62-0.79)
Cerebral infarction					
NRT	26,386	597	22.6	1	1
Bupropion	1,628	12	7.4	0.33 (0.18-0.58)	0.54 (0.30-0.95)
Varenicline	12,753	96	7.5	0.33 (0.27-0.41)	0.53 (0.42-0.65)
Heart failure					
NRT	26,452	189	7.1	1	1
Bupropion	1,629	3	1.8	0.26 (0.08-0.81)	0.45 (0.15-1.51)
Varenicline	12,763	27	2.1	0.30 (0.20-0.44)	0.52 (0.34-0.79)
Peripheral vascular disease					
NRT	26,436	282	10.7	1	1
Bupropion	1,628	11	6.8	0.63 (0.35-1.16)	0.99 (0.54-1.82)
Varenicline	12,754	88	6.9	0.65 (0.51-0.82)	0.92 (0.71-1.15)
Arrhythmia					
NRT	26,427	359	13.6	1	1
Bupropion	1,628	9	5.5	0.41 (0.21-0.79)	0.67 (0.35-1.30)
Varenicline	12,754	85	6.7	0.49 (0.39-0.62)	0.76 (0.60-0.97)
Depression					
NRT	25,761	5,165	200.5	1	1
Bupropion	1,601	208	129.9	0.65 (0.57-0.75)	0.70 (0.61-0.81)
Varenicline	12,601	1,291	102.5	0.51 (0.48-0.54)	0.58 (0.54-0.61)
Self-harm					
NRT	26,436	321	12.1	1	1
Bupropion	1,628	10	6.1	0.51 (0.27-0.95)	0.62 (0.33-1.17)
Varenicline	12,758	68	5.3	0.44 (0.34-0.57)	0.53 (0.41-0.69)

NRT = nicotine replacement therapy. *Adjusted for age, sex, socio-economic status, Strategic Health Authority of the general practice, comorbidities (i.e., prior recordings of COPD, diabetes, peptic ulcer disease, renal disease, rheumatological disease, or cancer), alcohol misuse, and any recordings of the neuropsychiatric and cardiovascular events of interest that occurred prior to the patient's entry date to the cohort.

Table E9: Hazard Ratios (95%CI) of Events during 3 Months Follow-up in the Propensity Score Matched Samples

Event	Hazard ratio (95% CI)	
	**Bupropion vs. NRT** (N=12,786)	**Varenicline vs. NRT** (N=100,326)
Ischemic heart disease	0.55 (0.32-0.96)	0.76 (0.66-0.88)
Cerebral infarction	0.53 (0.24-1.19)	0.48 (0.36-0.64)
Heart failure	0.25 (0.03-2.24)	0.60 (0.35-1.06)
Peripheral vascular disease	2.50 (0.78-7.97)	1.05 (0.77-1.44)
Arrhythmia	0.57 (0.24-1.36)	0.84 (0.61-1.15)
Depression	0.71 (0.60-0.86)	0.56 (0.52-0.60)
Self-harm	0.62 (0.28-1.38)	0.56 (0.41-0.76)

NRT = nicotine replacement therapy.

Table E10: Hazard ratio (95%CI) for ischemic heart disease during 3 months follow-up in users of varenicline vs. NRT, adjusted for an unmeasured binary confounder with a hazard ratio of 3

P1 / P0	0.0	0.1	0.2	0.3	0.4	0.5	0.6	0.7	0.8	0.9	1.0
0.0	0.69 (0.62, 0.79)	0.83 (0.74, 0.95)	0.97 (0.87, 1.11)	1.1 (0.99, 1.26)	1.24 (1.12, 1.42)	1.38 (1.24, 1.58)	1.52 (1.36, 1.74)	1.66 (1.49, 1.9)	1.79 (1.61, 2.05)	1.93 (1.74, 2.21)	2.07 (1.86, 2.37)
0.1	0.58 (0.52, 0.66)	0.69 (0.62, 0.79)	0.81 (0.72, 0.92)	0.92 (0.83, 1.05)	1.04 (0.93, 1.19)	1.15 (1.03, 1.32)	1.27 (1.14, 1.45)	1.38 (1.24, 1.58)	1.5 (1.34, 1.71)	1.61 (1.45, 1.84)	1.73 (1.55, 1.98)
0.2	0.49 (0.44, 0.56)	0.59 (0.53, 0.68)	0.69 (0.62, 0.79)	0.79 (0.71, 0.9)	0.89 (0.8, 1.02)	0.99 (0.89, 1.13)	1.08 (0.97, 1.24)	1.18 (1.06, 1.35)	1.28 (1.15, 1.47)	1.38 (1.24, 1.58)	1.48 (1.33, 1.69)
0.3	0.43 (0.39, 0.49)	0.52 (0.47, 0.59)	0.6 (0.54, 0.69)	0.69 (0.62, 0.79)	0.78 (0.7, 0.89)	0.86 (0.78, 0.99)	0.95 (0.85, 1.09)	1.04 (0.93, 1.19)	1.12 (1.01, 1.28)	1.21 (1.09, 1.38)	1.29 (1.16, 1.48)
0.4	0.38 (0.34, 0.44)	0.46 (0.41, 0.53)	0.54 (0.48, 0.61)	0.61 (0.55, 0.7)	0.69 (0.62, 0.79)	0.77 (0.69, 0.88)	0.84 (0.76, 0.97)	0.92 (0.83, 1.05)	1 (0.9, 1.14)	1.07 (0.96, 1.23)	1.15 (1.03, 1.32)
0.5	0.35 (0.31, 0.4)	0.41 (0.37, 0.47)	0.48 (0.43, 0.55)	0.55 (0.5, 0.63)	0.62 (0.56, 0.71)	0.69 (0.62, 0.79)	0.76 (0.68, 0.87)	0.83 (0.74, 0.95)	0.9 (0.81, 1.03)	0.97 (0.87, 1.11)	1.04 (0.93, 1.19)
0.6	0.31 (0.28, 0.36)	0.38 (0.34, 0.43)	0.44 (0.39, 0.5)	0.5 (0.45, 0.57)	0.56 (0.51, 0.65)	0.63 (0.56, 0.72)	0.69 (0.62, 0.79)	0.75 (0.68, 0.86)	0.82 (0.73, 0.93)	0.88 (0.79, 1.01)	0.94 (0.85, 1.08)
0.7	0.29 (0.26, 0.33)	0.35 (0.31, 0.4)	0.4 (0.36, 0.46)	0.46 (0.41, 0.53)	0.52 (0.47, 0.59)	0.58 (0.52, 0.66)	0.63 (0.57, 0.72)	0.69 (0.62, 0.79)	0.75 (0.67, 0.86)	0.81 (0.72, 0.92)	0.86 (0.78, 0.99)
0.8	0.27 (0.24, 0.3)	0.32 (0.29, 0.36)	0.37 (0.33, 0.43)	0.42 (0.38, 0.49)	0.48 (0.43, 0.55)	0.53 (0.48, 0.61)	0.58 (0.52, 0.67)	0.64 (0.57, 0.73)	0.69 (0.62, 0.79)	0.74 (0.67, 0.85)	0.8 (0.72, 0.91)
0.9	0.25 (0.22, 0.28)	0.3 (0.27, 0.34)	0.35 (0.31, 0.4)	0.39 (0.35, 0.45)	0.44 (0.4, 0.51)	0.49 (0.44, 0.56)	0.54 (0.49, 0.62)	0.59 (0.53, 0.68)	0.64 (0.58, 0.73)	0.69 (0.62, 0.79)	0.74 (0.66, 0.85)
1.0	0.23 (0.21, 0.26)	0.28 (0.25, 0.32)	0.32 (0.29, 0.37)	0.37 (0.33, 0.42)	0.41 (0.37, 0.47)	0.46 (0.41, 0.53)	0.51 (0.45, 0.58)	0.55 (0.5, 0.63)	0.6 (0.54, 0.68)	0.64 (0.58, 0.74)	0.69 (0.62, 0.79)

This tables shows how the observed hazard ratio (diagonal line of cells filled with light blue) would change in the presence of an unmeasured confounder with a hazard ratio of 3 and different combinations of prevalence rates among the user groups. P1/P0 = prevalence of the unmeasured confounder among users of varenicline (P1) and NRT (P0). The cells filled with red mark the situations in which varenicline would be associated with a statistically significant increased hazard of the event. In addition, the cells filled with dark red mark the situations in which this hazard would also be clinically meaningful according to our study protocol (HR>=1.5). These calculations are based on: Lin et al. Biometrics 1998, 54(3), 948-963 (equation 2.9).

Table E11: Hazard ratio (95%CI) for cerebral infarction during 3 months follow-up in users of varenicline vs. NRT, adjusted for an unmeasured binary confounder with a hazard ratio of 3

P1 / P0	0.0	0.1	0.2	0.3	0.4	0.5	0.6	0.7	0.8	0.9	1.0
0.0	0.53 (0.42, 0.65)	0.64 (0.5, 0.78)	0.74 (0.59, 0.91)	0.85 (0.67, 1.04)	0.95 (0.76, 1.17)	1.06 (0.84, 1.3)	1.17 (0.92, 1.43)	1.27 (1.01, 1.56)	1.38 (1.09, 1.69)	1.48 (1.18, 1.82)	1.59 (1.26, 1.95)
0.1	0.44 (0.35, 0.54)	0.53 (0.42, 0.65)	0.62 (0.49, 0.76)	0.71 (0.56, 0.87)	0.8 (0.63, 0.98)	0.88 (0.7, 1.08)	0.97 (0.77, 1.19)	1.06 (0.84, 1.3)	1.15 (0.91, 1.41)	1.24 (0.98, 1.52)	1.33 (1.05, 1.63)
0.2	0.38 (0.3, 0.46)	0.45 (0.36, 0.56)	0.53 (0.42, 0.65)	0.61 (0.48, 0.74)	0.68 (0.54, 0.84)	0.76 (0.6, 0.93)	0.83 (0.66, 1.02)	0.91 (0.72, 1.11)	0.98 (0.78, 1.21)	1.06 (0.84, 1.3)	1.14 (0.9, 1.39)
0.3	0.33 (0.26, 0.41)	0.4 (0.32, 0.49)	0.46 (0.37, 0.57)	0.53 (0.42, 0.65)	0.6 (0.47, 0.73)	0.66 (0.53, 0.81)	0.73 (0.58, 0.89)	0.8 (0.63, 0.98)	0.86 (0.68, 1.06)	0.93 (0.74, 1.14)	0.99 (0.79, 1.22)
0.4	0.29 (0.23, 0.36)	0.35 (0.28, 0.43)	0.41 (0.33, 0.51)	0.47 (0.37, 0.58)	0.53 (0.42, 0.65)	0.59 (0.47, 0.72)	0.65 (0.51, 0.79)	0.71 (0.56, 0.87)	0.77 (0.61, 0.94)	0.82 (0.65, 1.01)	0.88 (0.7, 1.08)
0.5	0.27 (0.21, 0.33)	0.32 (0.25, 0.39)	0.37 (0.29, 0.46)	0.42 (0.34, 0.52)	0.48 (0.38, 0.59)	0.53 (0.42, 0.65)	0.58 (0.46, 0.72)	0.64 (0.5, 0.78)	0.69 (0.55, 0.85)	0.74 (0.59, 0.91)	0.8 (0.63, 0.98)
0.6	0.24 (0.19, 0.3)	0.29 (0.23, 0.35)	0.34 (0.27, 0.41)	0.39 (0.31, 0.47)	0.43 (0.34, 0.53)	0.48 (0.38, 0.59)	0.53 (0.42, 0.65)	0.58 (0.46, 0.71)	0.63 (0.5, 0.77)	0.67 (0.53, 0.83)	0.72 (0.57, 0.89)
0.7	0.22 (0.18, 0.27)	0.27 (0.21, 0.33)	0.31 (0.25, 0.38)	0.35 (0.28, 0.43)	0.4 (0.32, 0.49)	0.44 (0.35, 0.54)	0.49 (0.39, 0.6)	0.53 (0.42, 0.65)	0.57 (0.46, 0.7)	0.62 (0.49, 0.76)	0.66 (0.53, 0.81)
0.8	0.2 (0.16, 0.25)	0.24 (0.19, 0.3)	0.29 (0.23, 0.35)	0.33 (0.26, 0.4)	0.37 (0.29, 0.45)	0.41 (0.32, 0.5)	0.45 (0.36, 0.55)	0.49 (0.39, 0.6)	0.53 (0.42, 0.65)	0.57 (0.45, 0.7)	0.61 (0.48, 0.75)
0.9	0.19 (0.15, 0.23)	0.23 (0.18, 0.28)	0.27 (0.21, 0.33)	0.3 (0.24, 0.37)	0.34 (0.27, 0.42)	0.38 (0.3, 0.46)	0.42 (0.33, 0.51)	0.45 (0.36, 0.56)	0.49 (0.39, 0.6)	0.53 (0.42, 0.65)	0.57 (0.45, 0.7)
1.0	0.18 (0.14, 0.22)	0.21 (0.17, 0.26)	0.25 (0.2, 0.3)	0.28 (0.22, 0.35)	0.32 (0.25, 0.39)	0.35 (0.28, 0.43)	0.39 (0.31, 0.48)	0.42 (0.34, 0.52)	0.46 (0.36, 0.56)	0.49 (0.39, 0.61)	0.53 (0.42, 0.65)

This tables shows how the observed hazard ratio (diagonal line of cells filled with light blue) would change in the presence of an unmeasured confounder with a hazard ratio of 3 and different combinations of prevalence rates among the user groups. P1/P0 = prevalence of the unmeasured confounder among users of varenicline (P1) and NRT (P0). The cells filled with red mark the situations in which varenicline would be associated with a statistically significant increased hazard of the event. In addition, the cells filled with dark red mark the situations in which this hazard would also be clinically meaningful according to our study protocol (HR>=1.5). These calculations are based on: Lin et al. Biometrics 1998, 54(3), 948-963 (equation 2.9).

Table E12: Hazard ratio (95%CI) for heart failure during 3 months follow-up in users of varenicline vs. NRT, adjusted for an unmeasured binary confounder with a hazard ratio of 3

P1 / P0	0.0	0.1	0.2	0.3	0.4	0.5	0.6	0.7	0.8	0.9	1.0
0.0	0.52 (0.34, 0.79)	0.62 (0.41, 0.95)	0.73 (0.48, 1.11)	0.83 (0.54, 1.26)	0.94 (0.61, 1.42)	1.04 (0.68, 1.58)	1.14 (0.75, 1.74)	1.25 (0.82, 1.9)	1.35 (0.88, 2.05)	1.46 (0.95, 2.21)	1.56 (1.02, 2.37)
0.1	0.43 (0.28, 0.66)	0.52 (0.34, 0.79)	0.61 (0.4, 0.92)	0.69 (0.45, 1.05)	0.78 (0.51, 1.19)	0.87 (0.57, 1.32)	0.95 (0.62, 1.45)	1.04 (0.68, 1.58)	1.13 (0.74, 1.71)	1.21 (0.79, 1.84)	1.3 (0.85, 1.98)
0.2	0.37 (0.24, 0.56)	0.45 (0.29, 0.68)	0.52 (0.34, 0.79)	0.59 (0.39, 0.9)	0.67 (0.44, 1.02)	0.74 (0.49, 1.13)	0.82 (0.53, 1.24)	0.89 (0.58, 1.35)	0.97 (0.63, 1.47)	1.04 (0.68, 1.58)	1.11 (0.73, 1.69)
0.3	0.33 (0.21, 0.49)	0.39 (0.26, 0.59)	0.46 (0.3, 0.69)	0.52 (0.34, 0.79)	0.59 (0.38, 0.89)	0.65 (0.43, 0.99)	0.72 (0.47, 1.09)	0.78 (0.51, 1.19)	0.85 (0.55, 1.28)	0.91 (0.6, 1.38)	0.98 (0.64, 1.48)
0.4	0.29 (0.19, 0.44)	0.35 (0.23, 0.53)	0.4 (0.26, 0.61)	0.46 (0.3, 0.7)	0.52 (0.34, 0.79)	0.58 (0.38, 0.88)	0.64 (0.42, 0.97)	0.69 (0.45, 1.05)	0.75 (0.49, 1.14)	0.81 (0.53, 1.23)	0.87 (0.57, 1.32)
0.5	0.26 (0.17, 0.4)	0.31 (0.2, 0.47)	0.36 (0.24, 0.55)	0.42 (0.27, 0.63)	0.47 (0.31, 0.71)	0.52 (0.34, 0.79)	0.57 (0.37, 0.87)	0.62 (0.41, 0.95)	0.68 (0.44, 1.03)	0.73 (0.48, 1.11)	0.78 (0.51, 1.19)
0.6	0.24 (0.15, 0.36)	0.28 (0.19, 0.43)	0.33 (0.22, 0.5)	0.38 (0.25, 0.57)	0.43 (0.28, 0.65)	0.47 (0.31, 0.72)	0.52 (0.34, 0.79)	0.57 (0.37, 0.86)	0.61 (0.4, 0.93)	0.66 (0.43, 1.01)	0.71 (0.46, 1.08)
0.7	0.22 (0.14, 0.33)	0.26 (0.17, 0.4)	0.3 (0.2, 0.46)	0.35 (0.23, 0.53)	0.39 (0.26, 0.59)	0.43 (0.28, 0.66)	0.48 (0.31, 0.72)	0.52 (0.34, 0.79)	0.56 (0.37, 0.86)	0.61 (0.4, 0.92)	0.65 (0.43, 0.99)
0.8	0.2 (0.13, 0.3)	0.24 (0.16, 0.36)	0.28 (0.18, 0.43)	0.32 (0.21, 0.49)	0.36 (0.24, 0.55)	0.4 (0.26, 0.61)	0.44 (0.29, 0.67)	0.48 (0.31, 0.73)	0.52 (0.34, 0.79)	0.56 (0.37, 0.85)	0.6 (0.39, 0.91)
0.9	0.19 (0.12, 0.28)	0.22 (0.15, 0.34)	0.26 (0.17, 0.4)	0.3 (0.19, 0.45)	0.33 (0.22, 0.51)	0.37 (0.24, 0.56)	0.41 (0.27, 0.62)	0.45 (0.29, 0.68)	0.48 (0.32, 0.73)	0.52 (0.34, 0.79)	0.56 (0.36, 0.85)
1.0	0.17 (0.11, 0.26)	0.21 (0.14, 0.32)	0.24 (0.16, 0.37)	0.28 (0.18, 0.42)	0.31 (0.2, 0.47)	0.35 (0.23, 0.53)	0.38 (0.25, 0.58)	0.42 (0.27, 0.63)	0.45 (0.29, 0.68)	0.49 (0.32, 0.74)	0.52 (0.34, 0.79)

This tables shows how the observed hazard ratio (diagonal line of cells filled with light blue) would change in the presence of an unmeasured confounder with a hazard ratio of 3 and different combinations of prevalence rates among the user groups. P1/P0 = prevalence of the unmeasured confounder among users of varenicline (P1) and NRT (P0). The cells filled with red mark the situations in which varenicline would be associated with a statistically significant increased hazard of the event. In addition, the cells filled with dark red mark the situations in which this hazard would also be clinically meaningful according to our study protocol (HR>=1.5). These calculations are based on: Lin et al. Biometrics 1998, 54(3), 948-963 (equation 2.9).

Table E13: Hazard ratio (95%CI) for peripheral vascular disease during 3 months follow-up in users of varenicline vs. NRT, adjusted for an unmeasured binary confounder with a hazard ratio of 3

P1 / P0	0.0	0.1	0.2	0.3	0.4	0.5	0.6	0.7	0.8	0.9	1.0
0.0	0.92 (0.71, 1.15)	1.1 (0.85, 1.38)	1.29 (0.99, 1.61)	1.47 (1.14, 1.84)	1.66 (1.28, 2.07)	1.84 (1.42, 2.3)	2.02 (1.56, 2.53)	2.21 (1.7, 2.76)	2.39 (1.85, 2.99)	2.58 (1.99, 3.22)	2.76 (2.13, 3.45)
0.1	0.77 (0.59, 0.96)	0.92 (0.71, 1.15)	1.07 (0.83, 1.34)	1.23 (0.95, 1.53)	1.38 (1.07, 1.73)	1.53 (1.18, 1.92)	1.69 (1.3, 2.11)	1.84 (1.42, 2.3)	1.99 (1.54, 2.49)	2.15 (1.66, 2.68)	2.3 (1.78, 2.88)
0.2	0.66 (0.51, 0.82)	0.79 (0.61, 0.99)	0.92 (0.71, 1.15)	1.05 (0.81, 1.31)	1.18 (0.91, 1.48)	1.31 (1.01, 1.64)	1.45 (1.12, 1.81)	1.58 (1.22, 1.97)	1.71 (1.32, 2.14)	1.84 (1.42, 2.3)	1.97 (1.52, 2.46)
0.3	0.58 (0.44, 0.72)	0.69 (0.53, 0.86)	0.81 (0.62, 1.01)	0.92 (0.71, 1.15)	1.04 (0.8, 1.29)	1.15 (0.89, 1.44)	1.27 (0.98, 1.58)	1.38 (1.07, 1.73)	1.5 (1.15, 1.87)	1.61 (1.24, 2.01)	1.73 (1.33, 2.16)
0.4	0.51 (0.39, 0.64)	0.61 (0.47, 0.77)	0.72 (0.55, 0.89)	0.82 (0.63, 1.02)	0.92 (0.71, 1.15)	1.02 (0.79, 1.28)	1.12 (0.87, 1.41)	1.23 (0.95, 1.53)	1.33 (1.03, 1.66)	1.43 (1.1, 1.79)	1.53 (1.18, 1.92)
0.5	0.46 (0.36, 0.58)	0.55 (0.43, 0.69)	0.64 (0.5, 0.81)	0.74 (0.57, 0.92)	0.83 (0.64, 1.04)	0.92 (0.71, 1.15)	1.01 (0.78, 1.27)	1.1 (0.85, 1.38)	1.2 (0.92, 1.5)	1.29 (0.99, 1.61)	1.38 (1.07, 1.73)
0.6	0.42 (0.32, 0.52)	0.5 (0.39, 0.63)	0.59 (0.45, 0.73)	0.67 (0.52, 0.84)	0.75 (0.58, 0.94)	0.84 (0.65, 1.05)	0.92 (0.71, 1.15)	1 (0.77, 1.25)	1.09 (0.84, 1.36)	1.17 (0.9, 1.46)	1.25 (0.97, 1.57)
0.7	0.38 (0.3, 0.48)	0.46 (0.36, 0.58)	0.54 (0.41, 0.67)	0.61 (0.47, 0.77)	0.69 (0.53, 0.86)	0.77 (0.59, 0.96)	0.84 (0.65, 1.05)	0.92 (0.71, 1.15)	1 (0.77, 1.25)	1.07 (0.83, 1.34)	1.15 (0.89, 1.44)
0.8	0.35 (0.27, 0.44)	0.42 (0.33, 0.53)	0.5 (0.38, 0.62)	0.57 (0.44, 0.71)	0.64 (0.49, 0.8)	0.71 (0.55, 0.88)	0.78 (0.6, 0.97)	0.85 (0.66, 1.06)	0.92 (0.71, 1.15)	0.99 (0.76, 1.24)	1.06 (0.82, 1.33)
0.9	0.33 (0.25, 0.41)	0.39 (0.3, 0.49)	0.46 (0.36, 0.58)	0.53 (0.41, 0.66)	0.59 (0.46, 0.74)	0.66 (0.51, 0.82)	0.72 (0.56, 0.9)	0.79 (0.61, 0.99)	0.85 (0.66, 1.07)	0.92 (0.71, 1.15)	0.99 (0.76, 1.23)
1.0	0.31 (0.24, 0.38)	0.37 (0.28, 0.46)	0.43 (0.33, 0.54)	0.49 (0.38, 0.61)	0.55 (0.43, 0.69)	0.61 (0.47, 0.77)	0.67 (0.52, 0.84)	0.74 (0.57, 0.92)	0.8 (0.62, 1)	0.86 (0.66, 1.07)	0.92 (0.71, 1.15)

This tables shows how the observed hazard ratio (diagonal line of cells filled with light blue) would change in the presence of an unmeasured confounder with a hazard ratio of 3 and different combinations of prevalence rates among the user groups. P1/P0 = prevalence of the unmeasured confounder among users of varenicline (P1) and NRT (P0). The cells filled with red mark the situations in which varenicline would be associated with a statistically significant increased hazard of the event. In addition, the cells filled with dark red mark the situations in which this hazard would also be clinically meaningful according to our study protocol (HR>=1.5). These calculations are based on: Lin et al. Biometrics 1998, 54(3), 948-963 (equation 2.9).

Table E14: Hazard ratio (95%CI) for arrhythmia during 3 months follow-up in users of varenicline vs. NRT, adjusted for an unmeasured binary confounder with a hazard ratio of 3

P1 / P0	0.0	0.1	0.2	0.3	0.4	0.5	0.6	0.7	0.8	0.9	1.0
0.0	0.76 (0.6, 0.97)	0.91 (0.72, 1.16)	1.06 (0.84, 1.36)	1.22 (0.96, 1.55)	1.37 (1.08, 1.75)	1.52 (1.2, 1.94)	1.67 (1.32, 2.13)	1.82 (1.44, 2.33)	1.98 (1.56, 2.52)	2.13 (1.68, 2.72)	2.28 (1.8, 2.91)
0.1	0.63 (0.5, 0.81)	0.76 (0.6, 0.97)	0.89 (0.7, 1.13)	1.01 (0.8, 1.29)	1.14 (0.9, 1.46)	1.27 (1, 1.62)	1.39 (1.1, 1.78)	1.52 (1.2, 1.94)	1.65 (1.3, 2.1)	1.77 (1.4, 2.26)	1.9 (1.5, 2.43)
0.2	0.54 (0.43, 0.69)	0.65 (0.51, 0.83)	0.76 (0.6, 0.97)	0.87 (0.69, 1.11)	0.98 (0.77, 1.25)	1.09 (0.86, 1.39)	1.19 (0.94, 1.52)	1.3 (1.03, 1.66)	1.41 (1.11, 1.8)	1.52 (1.2, 1.94)	1.63 (1.29, 2.08)
0.3	0.48 (0.38, 0.61)	0.57 (0.45, 0.73)	0.67 (0.53, 0.85)	0.76 (0.6, 0.97)	0.86 (0.68, 1.09)	0.95 (0.75, 1.21)	1.05 (0.83, 1.33)	1.14 (0.9, 1.46)	1.24 (0.98, 1.58)	1.33 (1.05, 1.7)	1.43 (1.13, 1.82)
0.4	0.42 (0.33, 0.54)	0.51 (0.4, 0.65)	0.59 (0.47, 0.75)	0.68 (0.53, 0.86)	0.76 (0.6, 0.97)	0.84 (0.67, 1.08)	0.93 (0.73, 1.19)	1.01 (0.8, 1.29)	1.1 (0.87, 1.4)	1.18 (0.93, 1.51)	1.27 (1, 1.62)
0.5	0.38 (0.3, 0.49)	0.46 (0.36, 0.58)	0.53 (0.42, 0.68)	0.61 (0.48, 0.78)	0.68 (0.54, 0.87)	0.76 (0.6, 0.97)	0.84 (0.66, 1.07)	0.91 (0.72, 1.16)	0.99 (0.78, 1.26)	1.06 (0.84, 1.36)	1.14 (0.9, 1.46)
0.6	0.35 (0.27, 0.44)	0.41 (0.33, 0.53)	0.48 (0.38, 0.62)	0.55 (0.44, 0.71)	0.62 (0.49, 0.79)	0.69 (0.55, 0.88)	0.76 (0.6, 0.97)	0.83 (0.65, 1.06)	0.9 (0.71, 1.15)	0.97 (0.76, 1.23)	1.04 (0.82, 1.32)
0.7	0.32 (0.25, 0.4)	0.38 (0.3, 0.49)	0.44 (0.35, 0.57)	0.51 (0.4, 0.65)	0.57 (0.45, 0.73)	0.63 (0.5, 0.81)	0.7 (0.55, 0.89)	0.76 (0.6, 0.97)	0.82 (0.65, 1.05)	0.89 (0.7, 1.13)	0.95 (0.75, 1.21)
0.8	0.29 (0.23, 0.37)	0.35 (0.28, 0.45)	0.41 (0.32, 0.52)	0.47 (0.37, 0.6)	0.53 (0.42, 0.67)	0.58 (0.46, 0.75)	0.64 (0.51, 0.82)	0.7 (0.55, 0.9)	0.76 (0.6, 0.97)	0.82 (0.65, 1.04)	0.88 (0.69, 1.12)
0.9	0.27 (0.21, 0.35)	0.33 (0.26, 0.42)	0.38 (0.3, 0.49)	0.43 (0.34, 0.55)	0.49 (0.39, 0.62)	0.54 (0.43, 0.69)	0.6 (0.47, 0.76)	0.65 (0.51, 0.83)	0.71 (0.56, 0.9)	0.76 (0.6, 0.97)	0.81 (0.64, 1.04)
1.0	0.25 (0.2, 0.32)	0.3 (0.24, 0.39)	0.35 (0.28, 0.45)	0.41 (0.32, 0.52)	0.46 (0.36, 0.58)	0.51 (0.4, 0.65)	0.56 (0.44, 0.71)	0.61 (0.48, 0.78)	0.66 (0.52, 0.84)	0.71 (0.56, 0.91)	0.76 (0.6, 0.97)

This tables shows how the observed hazard ratio (diagonal line of cells filled with light blue) would change in the presence of an unmeasured confounder with a hazard ratio of 3 and different combinations of prevalence rates among the user groups. P1/P0 = prevalence of the unmeasured confounder among users of varenicline (P1) and NRT (P0). The cells filled with red mark the situations in which varenicline would be associated with a statistically significant increased hazard of the event. In addition, the cells filled with dark red mark the situations in which this hazard would also be clinically meaningful according to our study protocol (HR>=1.5). These calculations are based on: Lin et al. Biometrics 1998, 54(3), 948-963 (equation 2.9).

Table E15: Hazard ratio (95%CI) for depression during 3 months follow-up in users of varenicline vs. NRT, adjusted for an unmeasured binary confounder with a hazard ratio of 3

P1 / P0	0.0	0.1	0.2	0.3	0.4	0.5	0.6	0.7	0.8	0.9	1.0
0.0	0.58 (0.54, 0.61)	0.7 (0.65, 0.73)	0.81 (0.76, 0.85)	0.93 (0.86, 0.98)	1.04 (0.97, 1.1)	1.16 (1.08, 1.22)	1.28 (1.19, 1.34)	1.39 (1.3, 1.46)	1.51 (1.4, 1.59)	1.62 (1.51, 1.71)	1.74 (1.62, 1.83)
0.1	0.48 (0.45, 0.51)	0.58 (0.54, 0.61)	0.68 (0.63, 0.71)	0.77 (0.72, 0.81)	0.87 (0.81, 0.92)	0.97 (0.9, 1.02)	1.06 (0.99, 1.12)	1.16 (1.08, 1.22)	1.26 (1.17, 1.32)	1.35 (1.26, 1.42)	1.45 (1.35, 1.53)
0.2	0.41 (0.39, 0.44)	0.5 (0.46, 0.52)	0.58 (0.54, 0.61)	0.66 (0.62, 0.7)	0.75 (0.69, 0.78)	0.83 (0.77, 0.87)	0.91 (0.85, 0.96)	0.99 (0.93, 1.05)	1.08 (1, 1.13)	1.16 (1.08, 1.22)	1.24 (1.16, 1.31)
0.3	0.36 (0.34, 0.38)	0.44 (0.41, 0.46)	0.51 (0.47, 0.53)	0.58 (0.54, 0.61)	0.65 (0.61, 0.69)	0.73 (0.68, 0.76)	0.8 (0.74, 0.84)	0.87 (0.81, 0.92)	0.94 (0.88, 0.99)	1.02 (0.95, 1.07)	1.09 (1.01, 1.14)
0.4	0.32 (0.3, 0.34)	0.39 (0.36, 0.41)	0.45 (0.42, 0.47)	0.52 (0.48, 0.54)	0.58 (0.54, 0.61)	0.64 (0.6, 0.68)	0.71 (0.66, 0.75)	0.77 (0.72, 0.81)	0.84 (0.78, 0.88)	0.9 (0.84, 0.95)	0.97 (0.9, 1.02)
0.5	0.29 (0.27, 0.31)	0.35 (0.32, 0.37)	0.41 (0.38, 0.43)	0.46 (0.43, 0.49)	0.52 (0.49, 0.55)	0.58 (0.54, 0.61)	0.64 (0.59, 0.67)	0.7 (0.65, 0.73)	0.75 (0.7, 0.79)	0.81 (0.76, 0.85)	0.87 (0.81, 0.92)
0.6	0.26 (0.25, 0.28)	0.32 (0.29, 0.33)	0.37 (0.34, 0.39)	0.42 (0.39, 0.44)	0.47 (0.44, 0.5)	0.53 (0.49, 0.55)	0.58 (0.54, 0.61)	0.63 (0.59, 0.67)	0.69 (0.64, 0.72)	0.74 (0.69, 0.78)	0.79 (0.74, 0.83)
0.7	0.24 (0.23, 0.25)	0.29 (0.27, 0.31)	0.34 (0.32, 0.36)	0.39 (0.36, 0.41)	0.44 (0.41, 0.46)	0.48 (0.45, 0.51)	0.53 (0.5, 0.56)	0.58 (0.54, 0.61)	0.63 (0.59, 0.66)	0.68 (0.63, 0.71)	0.73 (0.68, 0.76)
0.8	0.22 (0.21, 0.23)	0.27 (0.25, 0.28)	0.31 (0.29, 0.33)	0.36 (0.33, 0.38)	0.4 (0.37, 0.42)	0.45 (0.42, 0.47)	0.49 (0.46, 0.52)	0.54 (0.5, 0.56)	0.58 (0.54, 0.61)	0.62 (0.58, 0.66)	0.67 (0.62, 0.7)
0.9	0.21 (0.19, 0.22)	0.25 (0.23, 0.26)	0.29 (0.27, 0.31)	0.33 (0.31, 0.35)	0.37 (0.35, 0.39)	0.41 (0.39, 0.44)	0.46 (0.42, 0.48)	0.5 (0.46, 0.52)	0.54 (0.5, 0.57)	0.58 (0.54, 0.61)	0.62 (0.58, 0.65)
1.0	0.19 (0.18, 0.2)	0.23 (0.22, 0.24)	0.27 (0.25, 0.28)	0.31 (0.29, 0.33)	0.35 (0.32, 0.37)	0.39 (0.36, 0.41)	0.43 (0.4, 0.45)	0.46 (0.43, 0.49)	0.5 (0.47, 0.53)	0.54 (0.5, 0.57)	0.58 (0.54, 0.61)

This tables shows how the observed hazard ratio (diagonal line of cells filled with light blue) would change in the presence of an unmeasured confounder with a hazard ratio of 3 and different combinations of prevalence rates among the user groups. P1/P0 = prevalence of the unmeasured confounder among users of varenicline (P1) and NRT (P0). The cells filled with red mark the situations in which varenicline would be associated with a statistically significant increased hazard of the event. In addition, the cells filled with dark red mark the situations in which this hazard would also be clinically meaningful according to our study protocol (HR>=1.5). These calculations are based on: Lin et al. Biometrics 1998, 54(3), 948-963 (equation 2.9).

Table E16: Hazard ratio (95%CI) for self-harm during 3 months follow-up in users of varenicline vs. NRT, adjusted for an unmeasured binary confounder with a hazard ratio of 3

P1 / P0	0.0	0.1	0.2	0.3	0.4	0.5	0.6	0.7	0.8	0.9	1.0
0.0	0.53 (0.41, 0.69)	0.64 (0.49, 0.83)	0.74 (0.57, 0.97)	0.85 (0.66, 1.1)	0.95 (0.74, 1.24)	1.06 (0.82, 1.38)	1.17 (0.9, 1.52)	1.27 (0.98, 1.66)	1.38 (1.07, 1.79)	1.48 (1.15, 1.93)	1.59 (1.23, 2.07)
0.1	0.44 (0.34, 0.58)	0.53 (0.41, 0.69)	0.62 (0.48, 0.81)	0.71 (0.55, 0.92)	0.8 (0.62, 1.04)	0.88 (0.68, 1.15)	0.97 (0.75, 1.27)	1.06 (0.82, 1.38)	1.15 (0.89, 1.5)	1.24 (0.96, 1.61)	1.33 (1.03, 1.73)
0.2	0.38 (0.29, 0.49)	0.45 (0.35, 0.59)	0.53 (0.41, 0.69)	0.61 (0.47, 0.79)	0.68 (0.53, 0.89)	0.76 (0.59, 0.99)	0.83 (0.64, 1.08)	0.91 (0.7, 1.18)	0.98 (0.76, 1.28)	1.06 (0.82, 1.38)	1.14 (0.88, 1.48)
0.3	0.33 (0.26, 0.43)	0.4 (0.31, 0.52)	0.46 (0.36, 0.6)	0.53 (0.41, 0.69)	0.6 (0.46, 0.78)	0.66 (0.51, 0.86)	0.73 (0.56, 0.95)	0.8 (0.62, 1.04)	0.86 (0.67, 1.12)	0.93 (0.72, 1.21)	0.99 (0.77, 1.29)
0.4	0.29 (0.23, 0.38)	0.35 (0.27, 0.46)	0.41 (0.32, 0.54)	0.47 (0.36, 0.61)	0.53 (0.41, 0.69)	0.59 (0.46, 0.77)	0.65 (0.5, 0.84)	0.71 (0.55, 0.92)	0.77 (0.59, 1)	0.82 (0.64, 1.07)	0.88 (0.68, 1.15)
0.5	0.27 (0.21, 0.35)	0.32 (0.25, 0.41)	0.37 (0.29, 0.48)	0.42 (0.33, 0.55)	0.48 (0.37, 0.62)	0.53 (0.41, 0.69)	0.58 (0.45, 0.76)	0.64 (0.49, 0.83)	0.69 (0.53, 0.9)	0.74 (0.57, 0.97)	0.8 (0.62, 1.04)
0.6	0.24 (0.19, 0.31)	0.29 (0.22, 0.38)	0.34 (0.26, 0.44)	0.39 (0.3, 0.5)	0.43 (0.34, 0.56)	0.48 (0.37, 0.63)	0.53 (0.41, 0.69)	0.58 (0.45, 0.75)	0.63 (0.48, 0.82)	0.67 (0.52, 0.88)	0.72 (0.56, 0.94)
0.7	0.22 (0.17, 0.29)	0.27 (0.21, 0.35)	0.31 (0.24, 0.4)	0.35 (0.27, 0.46)	0.4 (0.31, 0.52)	0.44 (0.34, 0.58)	0.49 (0.38, 0.63)	0.53 (0.41, 0.69)	0.57 (0.44, 0.75)	0.62 (0.48, 0.81)	0.66 (0.51, 0.86)
0.8	0.2 (0.16, 0.27)	0.24 (0.19, 0.32)	0.29 (0.22, 0.37)	0.33 (0.25, 0.42)	0.37 (0.28, 0.48)	0.41 (0.32, 0.53)	0.45 (0.35, 0.58)	0.49 (0.38, 0.64)	0.53 (0.41, 0.69)	0.57 (0.44, 0.74)	0.61 (0.47, 0.8)
0.9	0.19 (0.15, 0.25)	0.23 (0.18, 0.3)	0.27 (0.21, 0.35)	0.3 (0.23, 0.39)	0.34 (0.26, 0.44)	0.38 (0.29, 0.49)	0.42 (0.32, 0.54)	0.45 (0.35, 0.59)	0.49 (0.38, 0.64)	0.53 (0.41, 0.69)	0.57 (0.44, 0.74)
1.0	0.18 (0.14, 0.23)	0.21 (0.16, 0.28)	0.25 (0.19, 0.32)	0.28 (0.22, 0.37)	0.32 (0.25, 0.41)	0.35 (0.27, 0.46)	0.39 (0.3, 0.51)	0.42 (0.33, 0.55)	0.46 (0.36, 0.6)	0.49 (0.38, 0.64)	0.53 (0.41, 0.69)

This tables shows how the observed hazard ratio (diagonal line of cells filled with light blue) would change in the presence of an unmeasured confounder with a hazard ratio of 3 and different combinations of prevalence rates among the user groups. P1/P0 = prevalence of the unmeasured confounder among users of varenicline (P1) and NRT Rx (P0). The cells filled with red mark the situations in which varenicline would be associated with a statistically significant increased hazard of the event. In addition, the cells filled with dark red mark the situations in which this hazard would also be clinically meaningful according to our study protocol (HR>=1.5). These calculations are based on: Lin et al. Biometrics 1998, 54(3), 948-963 (equation 2.9).

R code

Reference to study protocol: Kotz D, Simpson C, Viechtbauer W, et al. Cardiovascular and neuropsychiatric safety of varenicline and bupropion compared with nicotine replacement therapy for smoking cessation: study protocol of a retrospective cohort study using the QResearch general practice database. BMJ Open 2014; 4

```
##########################################################################################

rm(list=ls()) #clear the workspace

### install packages, load libraries
#print(.packages()) #to list loaded packages
if(!require("ROCR"))
  install.packages("ROCR")
if(!require("MatchIt"))
  install.packages("MatchIt")
library(survival)
library(ROCR)
library(MatchIt)

### read in dat_final.csv
dat <- read.table("dat_final.csv", header=TRUE, sep=",", as.is=TRUE)

### coerce "med" into a factor with level N, B, and V (N is reference)
dat$med <- factor(dat$med, levels=c("N", "B", "V"))

### rename "sex1" variable to "sex"
names(dat)[which(names(dat) == "sex1")] <- "sex"

### coerce "sex" into a factor with level female and male (female is reference)
dat$sex <- factor(dat$sex, levels=c("female", "male"))

### create age deciles
dat$age.cat <- cut(dat$age, breaks=quantile(dat$age, prob=seq(0,1,.1)), include.lowest=TRUE)

### create age dummy
dat$age.cat <- ifelse(dat$age > median(dat$age), "older", "younger")

###Sample size#################################################################################

### number of patients with missing data / definition complete case sample
head(dat,1)
sum(is.na(dat$age))
sum(is.na(dat$sex))
sum(is.na(dat$townsend_quintile))
sum(is.na(dat$sha1))

### show data for patients that have missing values on these variables
#dat[is.na(dat$age),]
#dat[is.na(dat$sex),]
#dat[is.na(dat$townsend_quintile),]
#dat[is.na(dat$sha1),]

### filter out patients that have missing values on these variables
dat <- dat[!is.na(dat$age),]
dat <- dat[!is.na(dat$sex),]
```

```
dat <- dat[!is.na(dat$townsend_quintile),]
dat <- dat[!is.na(dat$sha1),]

### number of patients and patient-years per drug group
table(dat$med)

sum(dat$selfharm.day[dat$med == "N"])/365 #other way of coding: sum(dat[dat$med == "N","selfharm.day"])
sum(dat$selfharm.day[dat$med == "B"])/365
sum(dat$selfharm.day[dat$med == "V"])/365
sum(dat$depression.day[dat$med == "N"])/365
sum(dat$depression.day[dat$med == "B"])/365
sum(dat$depression.day[dat$med == "V"])/365
sum(dat$ihd.day[dat$med == "N"])/365
sum(dat$ihd.day[dat$med == "B"])/365
sum(dat$ihd.day[dat$med == "V"])/365
sum(dat$stroke.day[dat$med == "N"])/365
sum(dat$stroke.day[dat$med == "B"])/365
sum(dat$stroke.day[dat$med == "V"])/365
sum(dat$hf.day[dat$med == "N"])/365
sum(dat$hf.day[dat$med == "B"])/365
sum(dat$hf.day[dat$med == "V"])/365
sum(dat$pvd.day[dat$med == "N"])/365
sum(dat$pvd.day[dat$med == "B"])/365
sum(dat$pvd.day[dat$med == "V"])/365
sum(dat$arrhythmia.day[dat$med == "N"])/365
sum(dat$arrhythmia.day[dat$med == "B"])/365
sum(dat$arrhythmia.day[dat$med == "V"])/365

### baseline characteristics for categorical variables
table(dat$med, dat$sex)
prop.table(table(dat$med, dat$sex), margin=1)
table(dat$med, dat$sha1)
prop.table(table(dat$med, dat$sha1), margin=1)

table(dat$med, dat$copd)
prop.table(table(dat$med, dat$copd), margin=1)
table(dat$med, dat$diabetes.prior)
prop.table(table(dat$med, dat$diabetes.prior), margin=1)
table(dat$med, dat$ulcer.prior)
prop.table(table(dat$med, dat$ulcer.prior), margin=1)
table(dat$med, dat$renal.prior)
prop.table(table(dat$med, dat$renal.prior), margin=1)
table(dat$med, dat$rheuma.prior)
prop.table(table(dat$med, dat$rheuma.prior), margin=1)
table(dat$med, dat$cancer.prior)
prop.table(table(dat$med, dat$cancer.prior), margin=1)
table(dat$med, dat$alcohol.prior)
prop.table(table(dat$med, dat$alcohol.prior), margin=1)

table(dat$med, dat$selfharm.prior)
prop.table(table(dat$med, dat$selfharm.prior), margin=1)
table(dat$med, dat$depression.prior)
prop.table(table(dat$med, dat$depression.prior), margin=1)
table(dat$med, dat$ihd.prior)
prop.table(table(dat$med, dat$ihd.prior), margin=1)
table(dat$med, dat$stroke.prior)
prop.table(table(dat$med, dat$stroke.prior), margin=1)
```

```
table(dat$med, dat$hf.prior)
prop.table(table(dat$med, dat$hf.prior), margin=1)
table(dat$med, dat$pvd.prior)
prop.table(table(dat$med, dat$pvd.prior), margin=1)
table(dat$med, dat$arrhythmia.prior)
prop.table(table(dat$med, dat$arrhythmia.prior), margin=1)

### baseline characteristics for continuous variables
by(dat$age, dat$med, summary)
by(dat$age, dat$med, sd)
by(dat$townsend_quintile, dat$med, summary)
by(dat$townsend_quintile, dat$med, sd)

### testing baseline characteristics (all three groups) and pairwise comparisons for a dichotomous variable
res <- glm(sex ~ med, family=binomial, data=dat)
summary(res)
anova(res, test="LRT")
res <- glm(sex ~ relevel(med, ref="B"), family=binomial, data=dat)
summary(res)

res <- glm(copd ~ med, family=binomial, data=dat)
summary(res)
anova(res, test="LRT")
#confint (res) # 95%CI around model parameters
res <- glm(copd ~ relevel(med, ref="B"), family=binomial, data=dat)
summary(res)

res <- glm(diabetes.prior ~ med, family=binomial, data=dat)
summary(res)
anova(res, test="LRT")
res <- glm(diabetes.prior ~ relevel(med, ref="B"), family=binomial, data=dat)
summary(res)

res <- glm(ulcer.prior ~ med, family=binomial, data=dat)
summary(res)
anova(res, test="LRT")
res <- glm(ulcer.prior ~ relevel(med, ref="B"), family=binomial, data=dat)
summary(res)

res <- glm(renal.prior ~ med, family=binomial, data=dat)
summary(res)
anova(res, test="LRT")
res <- glm(renal.prior ~ relevel(med, ref="B"), family=binomial, data=dat)
summary(res)

res <- glm(rheuma.prior ~ med, family=binomial, data=dat)
summary(res)
anova(res, test="LRT")
res <- glm(rheuma.prior ~ relevel(med, ref="B"), family=binomial, data=dat)
summary(res)

res <- glm(cancer.prior ~ med, family=binomial, data=dat)
summary(res)
anova(res, test="LRT")
res <- glm(cancer.prior ~ relevel(med, ref="B"), family=binomial, data=dat)
summary(res)
```

```
res <- glm(alcohol.prior ~ med, family=binomial, data=dat)
summary(res)
anova(res, test="LRT")
res <- glm(alcohol.prior ~ relevel(med, ref="B"), family=binomial, data=dat)
summary(res)

res <- glm(selfharm.prior ~ med, family=binomial, data=dat)
summary(res)
anova(res, test="LRT")
res <- glm(selfharm.prior ~ relevel(med, ref="B"), family=binomial, data=dat)
summary(res)

res <- glm(depression.prior ~ med, family=binomial, data=dat)
summary(res)
anova(res, test="LRT")
res <- glm(depression.prior ~ relevel(med, ref="B"), family=binomial, data=dat)
summary(res)

res <- glm(ihd.prior ~ med, family=binomial, data=dat)
summary(res)
anova(res, test="LRT")
res <- glm(ihd.prior ~ relevel(med, ref="B"), family=binomial, data=dat)
summary(res)

res <- glm(stroke.prior ~ med, family=binomial, data=dat)
summary(res)
anova(res, test="LRT")
res <- glm(stroke.prior ~ relevel(med, ref="B"), family=binomial, data=dat)
summary(res)

res <- glm(hf.prior ~ med, family=binomial, data=dat)
summary(res)
anova(res, test="LRT")
res <- glm(hf.prior ~ relevel(med, ref="B"), family=binomial, data=dat)
summary(res)

res <- glm(pvd.prior ~ med, family=binomial, data=dat)
summary(res)
anova(res, test="LRT")
res <- glm(pvd.prior ~ relevel(med, ref="B"), family=binomial, data=dat)
summary(res)

res <- glm(arrhythmia.prior ~ med, family=binomial, data=dat)
summary(res)
anova(res, test="LRT")
res <- glm(arrhythmia.prior ~ relevel(med, ref="B"), family=binomial, data=dat)
summary(res)

### testing baseline characteristics (all three groups) and pairwise comparisons for a continuous variable
res <- lm(age ~ med, data=dat)
summary(res)
anova(res)
res <- lm(age ~ relevel(med, ref="B"), data=dat)
summary(res)

res <- lm(townsend_quintile ~ med, data=dat)
summary(res)
```

```
anova(res)
res <- lm(townsend_quintile ~ relevel(med, ref="B"), data=dat)
summary(res)

### number and proportion of patients that experienced the specific event
table(dat$med, dat$selfharm)
prop.table(table(dat$med, dat$selfharm), margin=1)
of low proportion (more exact)
table(dat$med, dat$depression)
prop.table(table(dat$med, dat$depression), margin=1)
table(dat$med, dat$ihd)
prop.table(table(dat$med, dat$ihd), margin=1)
table(dat$med, dat$stroke)
prop.table(table(dat$med, dat$stroke), margin=1)
table(dat$med, dat$hf)
prop.table(table(dat$med, dat$hf), margin=1)
table(dat$med, dat$pvd)
prop.table(table(dat$med, dat$pvd), margin=1)
table(dat$med, dat$arrhythmia)
prop.table(table(dat$med, dat$arrhythmia), margin=1)

###Survival analyses#################################################################################

### survival analysis and Kaplan-Meier curves: selfharm

resp <- Surv(dat$selfharm.day, dat$selfharm)

ylow <- .90 #lower y-axis limit for KM curve

res <- survfit(resp ~ med, data=dat)
print(res, print.rmean=TRUE)

png(filename="fig_KM_selfharm.png", res=350, width=3000, height=2500, type="cairo") #to save as png file

plot(res, col=c("blue", "red", "green"), xlab="Days", ylab="Survival Proportion", xlim=c(0,186), ylim=c(ylow,1), xaxt="n")
axis(side=1, at=seq(0,180,20))
legend(x=3, y=ylow, legend=c("NRT Rx", "Bupropion", "Varenicline"), col=c("blue", "red", "green"), lty="solid", yjust=0,
bty="n")
title("Self-harm")

dev.off()

### survival analysis and Kaplan-Meier curves: depression

resp <- Surv(dat$depression.day, dat$depression)

ylow <- .90 #lower y-axis limit for KM curve

res <- survfit(resp ~ med, data=dat)
print(res, print.rmean=TRUE)

png(filename="fig_KM_depression.png", res=350, width=3000, height=2500, type="cairo") #to save as png file

plot(res, col=c("blue", "red", "green"), xlab="Days", ylab="Survival Proportion", xlim=c(0,186), ylim=c(ylow,1), xaxt="n")
axis(side=1, at=seq(0,180,20))
legend(x=3, y=ylow, legend=c("NRT Rx", "Bupropion", "Varenicline"), col=c("blue", "red", "green"), lty="solid", yjust=0,
bty="n")
```

```
title("Depression")

dev.off()

### survival analysis and Kaplan-Meier curves: ihd

resp <- Surv(dat$ihd.day, dat$ihd)

ylow <- .90 #lower y-axis limit for KM curve

res <- survfit(resp ~ med, data=dat)
print(res, print.rmean=TRUE)

png(filename="fig_KM_ihd.png", res=350, width=3000, height=2500, type="cairo") #to save as png file

plot(res, col=c("blue", "red", "green"), xlab="Days", ylab="Survival Proportion", xlim=c(0,186), ylim=c(ylow,1), xaxt="n")
axis(side=1, at=seq(0,180,20))
legend(x=3, y=ylow, legend=c("NRT Rx", "Bupropion", "Varenicline"), col=c("blue", "red", "green"), lty="solid", yjust=0,
bty="n")
title("Ischaemic heart disease")

dev.off()

### survival analysis and Kaplan-Meier curves: stroke

resp <- Surv(dat$stroke.day, dat$stroke)

ylow <- .90 #lower y-axis limit for KM curve

res <- survfit(resp ~ med, data=dat)
print(res, print.rmean=TRUE)

png(filename="fig_KM_stroke.png", res=350, width=3000, height=2500, type="cairo") #to save as png file

plot(res, col=c("blue", "red", "green"), xlab="Days", ylab="Survival Proportion", xlim=c(0,186), ylim=c(ylow,1), xaxt="n")
axis(side=1, at=seq(0,180,20))
legend(x=3, y=ylow, legend=c("NRT Rx", "Bupropion", "Varenicline"), col=c("blue", "red", "green"), lty="solid", yjust=0,
bty="n")
title("Stroke")

dev.off()

### survival analysis and Kaplan-Meier curves: hf

resp <- Surv(dat$hf.day, dat$hf)

ylow <- .90 #lower y-axis limit for KM curve

res <- survfit(resp ~ med, data=dat)
print(res, print.rmean=TRUE)

png(filename="fig_KM_hf.png", res=350, width=3000, height=2500, type="cairo") #to save as png file

plot(res, col=c("blue", "red", "green"), xlab="Days", ylab="Survival Proportion", xlim=c(0,186), ylim=c(ylow,1), xaxt="n")
axis(side=1, at=seq(0,180,20))
legend(x=3, y=ylow, legend=c("NRT Rx", "Bupropion", "Varenicline"), col=c("blue", "red", "green"), lty="solid", yjust=0,
bty="n")
```

```
title("Heart failure")

dev.off()

### survival analysis and Kaplan-Meier curves: pvd

resp <- Surv(dat$pvd.day, dat$pvd)

ylow <- .90 #lower y-axis limit for KM curve

res <- survfit(resp ~ med, data=dat)
print(res, print.rmean=TRUE)

png(filename="fig_KM_pvd.png", res=350, width=3000, height=2500, type="cairo") #to save as png file

plot(res, col=c("blue", "red", "green"), xlab="Days", ylab="Survival Proportion", xlim=c(0,186), ylim=c(ylow,1), xaxt="n")
axis(side=1, at=seq(0,180,20))
legend(x=3, y=ylow, legend=c("NRT Rx", "Bupropion", "Varenicline"), col=c("blue", "red", "green"), lty="solid", yjust=0,
bty="n")
title("Peripheral vascular disease")

dev.off()

### survival analysis and Kaplan-Meier curves: arrhythmia

resp <- Surv(dat$arrhythmia.day, dat$arrhythmia)

ylow <- .90 #lower y-axis limit for KM curve

res <- survfit(resp ~ med, data=dat)
print(res, print.rmean=TRUE)

png(filename="fig_KM_arrhythmia.png", res=350, width=3000, height=2500, type="cairo") #to save as png file

plot(res, col=c("blue", "red", "green"), xlab="Days", ylab="Survival Proportion", xlim=c(0,186), ylim=c(ylow,1), xaxt="n")
axis(side=1, at=seq(0,180,20))
legend(x=3, y=ylow, legend=c("NRT Rx", "Bupropion", "Varenicline"), col=c("blue", "red", "green"), lty="solid", yjust=0,
bty="n")
title("Cardiac arrhythmia")

dev.off()

### crude and adjusted Cox proportional hazard model, and test for interaction (med x sex): selfharm

resp <- Surv(dat$selfharm.day, dat$selfharm)

res <- coxph(resp ~ med, data=dat)
summary(res)
summary(coxph(resp ~ relevel(med, ref="B"), data=dat))
cox.zph(res) #GLOBAL p should not be p<.05

### crude and adjusted Cox proportional hazard model, and test for interaction (med x sex): depression

resp <- Surv(dat$depression.day, dat$depression)

res <- coxph(resp ~ med, data=dat)
summary(res)
```

```
summary(coxph(resp ~ relevel(med, ref="B"), data=dat))
cox.zph(res)

res <- coxph(resp ~ med + age + sex + townsend_quintile + sha1 + selfharm.prior + depression.prior + ihd.prior +
stroke.prior + hf.prior + pvd.prior + arrhythmia.prior + copd + diabetes.prior + ulcer.prior + renal.prior + rheuma.prior +
cancer.prior + alcohol.prior, data=dat)
summary(res)
summary(coxph(resp ~ relevel(med, ref="B") + age + sex + townsend_quintile + sha1 + selfharm.prior + depression.prior
+ ihd.prior + stroke.prior + hf.prior + pvd.prior + arrhythmia.prior + copd + diabetes.prior + ulcer.prior + renal.prior +
rheuma.prior + cancer.prior + alcohol.prior, data=dat))

res1 <- coxph(resp ~ med*sex + age + townsend_quintile + sha1 + selfharm.prior + depression.prior + ihd.prior +
stroke.prior + hf.prior + pvd.prior + arrhythmia.prior + copd + diabetes.prior + ulcer.prior + renal.prior + rheuma.prior +
cancer.prior + alcohol.prior, data=dat)
anova(res1, res)
summary(res1)
summary(coxph(resp ~ med*relevel(sex, ref="male") + age + townsend_quintile + sha1 + selfharm.prior +
depression.prior + ihd.prior + stroke.prior + hf.prior + pvd.prior + arrhythmia.prior + copd + diabetes.prior + ulcer.prior
+ renal.prior + rheuma.prior + cancer.prior + alcohol.prior, data=dat))

### crude and adjusted Cox proportional hazard model, and test for interaction (med x sex): ihd

resp <- Surv(dat$ihd.day, dat$ihd)

res <- coxph(resp ~ med, data=dat)
summary(res)
summary(coxph(resp ~ relevel(med, ref="B"), data=dat))
cox.zph(res)

res <- coxph(resp ~ med + age + sex + townsend_quintile + sha1 + selfharm.prior + depression.prior + ihd.prior +
stroke.prior + hf.prior + pvd.prior + arrhythmia.prior + copd + diabetes.prior + ulcer.prior + renal.prior + rheuma.prior +
cancer.prior + alcohol.prior, data=dat)
summary(res)
summary(coxph(resp ~ relevel(med, ref="B") + age + sex + townsend_quintile + sha1 + selfharm.prior + depression.prior
+ ihd.prior + stroke.prior + hf.prior + pvd.prior + arrhythmia.prior + copd + diabetes.prior + ulcer.prior + renal.prior +
rheuma.prior + cancer.prior + alcohol.prior, data=dat))

res1 <- coxph(resp ~ med*sex + age + townsend_quintile + sha1 + selfharm.prior + depression.prior + ihd.prior +
stroke.prior + hf.prior + pvd.prior + arrhythmia.prior + copd + diabetes.prior + ulcer.prior + renal.prior + rheuma.prior +
cancer.prior + alcohol.prior, data=dat)
anova(res1, res)
summary(res1)
summary(coxph(resp ~ med*relevel(sex, ref="male") + age + townsend_quintile + sha1 + selfharm.prior +
depression.prior + ihd.prior + stroke.prior + hf.prior + pvd.prior + arrhythmia.prior + copd + diabetes.prior + ulcer.prior
+ renal.prior + rheuma.prior + cancer.prior + alcohol.prior, data=dat))

### crude and adjusted Cox proportional hazard model, and test for interaction (med x sex): stroke

resp <- Surv(dat$stroke.day, dat$stroke)

res <- coxph(resp ~ med, data=dat)
summary(res)
summary(coxph(resp ~ relevel(med, ref="B"), data=dat))
cox.zph(res)
```

```
res <- coxph(resp ~ med + age + sex + townsend_quintile + sha1 + selfharm.prior + depression.prior + ihd.prior +
stroke.prior + hf.prior + pvd.prior + arrhythmia.prior + copd + diabetes.prior + ulcer.prior + renal.prior + rheuma.prior +
cancer.prior + alcohol.prior, data=dat)
summary(res)
summary(coxph(resp ~ relevel(med, ref="B") + age + sex + townsend_quintile + sha1 + selfharm.prior + depression.prior
+ ihd.prior + stroke.prior + hf.prior + pvd.prior + arrhythmia.prior + copd + diabetes.prior + ulcer.prior + renal.prior +
rheuma.prior + cancer.prior + alcohol.prior, data=dat))

res1 <- coxph(resp ~ med*sex + age + townsend_quintile + sha1 + selfharm.prior + depression.prior + ihd.prior +
stroke.prior + hf.prior + pvd.prior + arrhythmia.prior + copd + diabetes.prior + ulcer.prior + renal.prior + rheuma.prior +
cancer.prior + alcohol.prior, data=dat)
anova(res1, res)
summary(res1)
summary(coxph(resp ~ med*relevel(sex, ref="male") + age + townsend_quintile + sha1 + selfharm.prior +
depression.prior + ihd.prior + stroke.prior + hf.prior + pvd.prior + arrhythmia.prior + copd + diabetes.prior + ulcer.prior
+ renal.prior + rheuma.prior + cancer.prior + alcohol.prior, data=dat))

### crude and adjusted Cox proportional hazard model, and test for interaction (med x sex): hf

resp <- Surv(dat$hf.day, dat$hf)

res <- coxph(resp ~ med, data=dat)
summary(res)
summary(coxph(resp ~ relevel(med, ref="B"), data=dat))
cox.zph(res)

res <- coxph(resp ~ med + age + sex + townsend_quintile + sha1 + selfharm.prior + depression.prior + ihd.prior +
stroke.prior + hf.prior + pvd.prior + arrhythmia.prior + copd + diabetes.prior + ulcer.prior + renal.prior + rheuma.prior +
cancer.prior + alcohol.prior, data=dat)
summary(res)
summary(coxph(resp ~ relevel(med, ref="B") + age + sex + townsend_quintile + sha1 + selfharm.prior + depression.prior
+ ihd.prior + stroke.prior + hf.prior + pvd.prior + arrhythmia.prior + copd + diabetes.prior + ulcer.prior + renal.prior +
rheuma.prior + cancer.prior + alcohol.prior, data=dat))

res1 <- coxph(resp ~ med*sex + age + townsend_quintile + sha1 + selfharm.prior + depression.prior + ihd.prior +
stroke.prior + hf.prior + pvd.prior + arrhythmia.prior + copd + diabetes.prior + ulcer.prior + renal.prior + rheuma.prior +
cancer.prior + alcohol.prior, data=dat)
anova(res1, res)
summary(res1)
summary(coxph(resp ~ med*relevel(sex, ref="male") + age + townsend_quintile + sha1 + selfharm.prior +
depression.prior + ihd.prior + stroke.prior + hf.prior + pvd.prior + arrhythmia.prior + copd + diabetes.prior + ulcer.prior
+ renal.prior + rheuma.prior + cancer.prior + alcohol.prior, data=dat))

### crude and adjusted Cox proportional hazard model, and test for interaction (med x sex): pvd

resp <- Surv(dat$pvd.day, dat$pvd)

res <- coxph(resp ~ med, data=dat)
summary(res)
summary(coxph(resp ~ relevel(med, ref="B"), data=dat))
cox.zph(res)

res <- coxph(resp ~ med + age + sex + townsend_quintile + sha1 + selfharm.prior + depression.prior + ihd.prior +
stroke.prior + hf.prior + pvd.prior + arrhythmia.prior + copd + diabetes.prior + ulcer.prior + renal.prior + rheuma.prior +
cancer.prior + alcohol.prior, data=dat)
summary(res)
```

```
summary(coxph(resp ~ relevel(med, ref="B") + age + sex + townsend_quintile + sha1 + selfharm.prior + depression.prior
+ ihd.prior + stroke.prior + hf.prior + pvd.prior + arrhythmia.prior + copd + diabetes.prior + ulcer.prior + renal.prior +
rheuma.prior + cancer.prior + alcohol.prior, data=dat))

res1 <- coxph(resp ~ med*sex + age + townsend_quintile + sha1 + selfharm.prior + depression.prior + ihd.prior +
stroke.prior + hf.prior + pvd.prior + arrhythmia.prior + copd + diabetes.prior + ulcer.prior + renal.prior + rheuma.prior +
cancer.prior + alcohol.prior, data=dat)
anova(res1, res)
summary(res1)
summary(coxph(resp ~ med*relevel(sex, ref="male") + age + townsend_quintile + sha1 + selfharm.prior +
depression.prior + ihd.prior + stroke.prior + hf.prior + pvd.prior + arrhythmia.prior + copd + diabetes.prior + ulcer.prior
+ renal.prior + rheuma.prior + cancer.prior + alcohol.prior, data=dat))

### crude and adjusted Cox proportional hazard model, and test for interaction (med x sex): arrhythmia

resp <- Surv(dat$arrhythmia.day, dat$arrhythmia)

res <- coxph(resp ~ med, data=dat)
summary(res)
summary(coxph(resp ~ relevel(med, ref="B"), data=dat))
cox.zph(res)

res <- coxph(resp ~ med + age + sex + townsend_quintile + sha1 + selfharm.prior + depression.prior + ihd.prior +
stroke.prior + hf.prior + pvd.prior + arrhythmia.prior + copd + diabetes.prior + ulcer.prior + renal.prior + rheuma.prior +
cancer.prior + alcohol.prior, data=dat)
summary(res)
summary(coxph(resp ~ relevel(med, ref="B") + age + sex + townsend_quintile + sha1 + selfharm.prior + depression.prior
+ ihd.prior + stroke.prior + hf.prior + pvd.prior + arrhythmia.prior + copd + diabetes.prior + ulcer.prior + renal.prior +
rheuma.prior + cancer.prior + alcohol.prior, data=dat))

res1 <- coxph(resp ~ med*sex + age + townsend_quintile + sha1 + selfharm.prior + depression.prior + ihd.prior +
stroke.prior + hf.prior + pvd.prior + arrhythmia.prior + copd + diabetes.prior + ulcer.prior + renal.prior + rheuma.prior +
cancer.prior + alcohol.prior, data=dat)
anova(res1, res)
summary(res1)
summary(coxph(resp ~ med*relevel(sex, ref="male") + age + townsend_quintile + sha1 + selfharm.prior +
depression.prior + ihd.prior + stroke.prior + hf.prior + pvd.prior + arrhythmia.prior + copd + diabetes.prior + ulcer.prior
+ renal.prior + rheuma.prior + cancer.prior + alcohol.prior, data=dat))

###Propensity scores#############################################################################

### propensity score analysis for N vs. B - step 1: trim and match 1:1

datNB <- subset(dat, med == "N" | med == "B")
datNB$med <- factor(datNB$med, levels=c("N","B"))
str(datNB)
table(datNB$med)

datNB$medB <- ifelse(datNB$med == "B", 1, 0)
res <- glm(medB ~ age + sex + townsend_quintile + sha1 + selfharm.prior + depression.prior + ihd.prior + stroke.prior +
hf.prior + pvd.prior + arrhythmia.prior + copd + diabetes.prior + ulcer.prior + renal.prior + rheuma.prior + cancer.prior +
alcohol.prior, family=binomial, data=datNB)
preds <- predict(res, type="response") #preds = predicted probabilities = propensity scores
by(preds, datNB$med, summary)
nrow(datNB)
table(datNB$med)
```

```
### ROC curve and AUC
pred <- prediction(preds, datNB$medB)
perf <- performance(pred, measure = "tpr", x.measure = "fpr")
plot(perf, lwd=2)
abline(a=0, b=1, lty="dotted")
performance(pred, measure="auc")@"y.values"[[1]]

### trim away the highest propensity scores of the N group and the lowest scores of the B group
sel1 <- datNB$med == "B" | (datNB$med == "N" & preds < quantile(preds[datNB$med == "N"], .975))
sel2 <- datNB$med == "N" | (datNB$med == "B" & preds > quantile(preds[datNB$med == "B"], .025))
datNB <- datNB[sel1 & sel2,]
preds <- preds[sel1 & sel2]

by(preds, datNB$med, summary)
nrow(datNB)

datNB$preds <- preds

dat.temp <- datNB[,c("medB","preds")]
res <- matchit(medB ~ preds, data=dat.temp)

datNB <- datNB[is.element(rownames(datNB), c(row.names(res$match.matrix), c(res$match.matrix))),]

table(datNB$med)
by(datNB$preds, datNB$med, summary)

### propensity score analysis for N vs. B - step 2: Cox models for all events

resp <- Surv(datNB$selfharm.day, datNB$selfharm)
res <- coxph(resp ~ med, data=datNB)
summary(res)
cox.zph(res)

resp <- Surv(datNB$depression.day, datNB$depression)
res <- coxph(resp ~ med, data=datNB)
summary(res)
cox.zph(res)

resp <- Surv(datNB$ihd.day, datNB$ihd)
res <- coxph(resp ~ med, data=datNB)
summary(res)
cox.zph(res)

resp <- Surv(datNB$stroke.day, datNB$stroke)
res <- coxph(resp ~ med, data=datNB)
summary(res)
cox.zph(res)

resp <- Surv(datNB$hf.day, datNB$hf)
res <- coxph(resp ~ med, data=datNB)
summary(res)
cox.zph(res)

resp <- Surv(datNB$pvd.day, datNB$pvd)
res <- coxph(resp ~ med, data=datNB)
summary(res)
cox.zph(res)
```

```
resp <- Surv(datNB$arrhythmia.day, datNB$arrhythmia)
res <- coxph(resp ~ med, data=datNB)
summary(res)
cox.zph(res)

### propensity score analysis for N vs. V - step 1: trim and match 1:1

datVB <- subset(dat, med == "N" | med == "V")
datVB$med <- factor(datVB$med, levels=c("N","V"))
str(datVB)
nrow(datVB)
table(datVB$med)

datVB$medV <- ifelse(datVB$med == "V", 1, 0)
res <- glm(medV ~ age + sex + townsend_quintile + sha1 + selfharm.prior + depression.prior + ihd.prior + stroke.prior +
hf.prior + pvd.prior + arrhythmia.prior + copd + diabetes.prior + ulcer.prior + renal.prior + rheuma.prior + cancer.prior +
alcohol.prior, family=binomial, data=datVB)
preds <- predict(res, type="response") #preds = predicted probabilities = propensity scores
by(preds, datVB$med, summary)

### ROC curve and AUC
pred <- prediction(preds, datVB$medV)
perf <- performance(pred, measure = "tpr", x.measure = "fpr")
plot(perf, lwd=2)
abline(a=0, b=1, lty="dotted")
performance(pred, measure="auc")@"y.values"[[1]]

### trim away the highest propensity scores of the N group and the lowest scores of the B group
sel1 <- datVB$med == "V" | (datVB$med == "N" & preds < quantile(preds[datVB$med == "N"], .975))
sel2 <- datVB$med == "N" | (datVB$med == "V" & preds > quantile(preds[datVB$med == "V"], .025))
datVB <- datVB[sel1 & sel2,]
preds <- preds[sel1 & sel2]

by(preds, datVB$med, summary)
nrow(datVB)

datVB$preds <- preds

dat.temp <- datVB[,c("medV","preds")]
res <- matchit(medV ~ preds, data=dat.temp)

datVB <- datVB[is.element(rownames(datVB), c(row.names(res$match.matrix), c(res$match.matrix))),]

table(datVB$med)
by(datVB$preds, datVB$med, summary)

### propensity score analysis for N vs. V - step 2: Cox models for all events

resp <- Surv(datVB$selfharm.day, datVB$selfharm)
res <- coxph(resp ~ med, data=datVB)
summary(res)
cox.zph(res)

resp <- Surv(datVB$depression.day, datVB$depression)
res <- coxph(resp ~ med, data=datVB)
summary(res)
```

```
cox.zph(res)

resp <- Surv(datVB$ihd.day, datVB$ihd)
res <- coxph(resp ~ med, data=datVB)
summary(res)
cox.zph(res)

resp <- Surv(datVB$stroke.day, datVB$stroke)
res <- coxph(resp ~ med, data=datVB)
summary(res)
cox.zph(res)

resp <- Surv(datVB$hf.day, datVB$hf)
res <- coxph(resp ~ med, data=datVB)
summary(res)
cox.zph(res)

resp <- Surv(datVB$pvd.day, datVB$pvd)
res <- coxph(resp ~ med, data=datVB)
summary(res)
cox.zph(res)

resp <- Surv(datVB$arrhythmia.day, datVB$arrhythmia)
res <- coxph(resp ~ med, data=datVB)
summary(res)
cox.zph(res)

###Sub-group analyses males/females##################################################################

### create subset of male/female patients

datF <- subset(dat, sex=="female")
datM <- subset(dat, sex=="male")

### adjusted Cox proportional hazard model: depression in males and females separately

resp <- Surv(datM$depression.day, datM$depression)
res <- coxph(resp ~ med + age + townsend_quintile + sha1 + selfharm.prior + depression.prior + ihd.prior + stroke.prior
+ hf.prior + pvd.prior + arrhythmia.prior + copd + diabetes.prior + ulcer.prior + renal.prior + rheuma.prior + cancer.prior
+ alcohol.prior, data=datM)
summary(res)
summary(coxph(resp ~ relevel(med, ref="B") + age + townsend_quintile + sha1 + selfharm.prior + depression.prior +
ihd.prior + stroke.prior + hf.prior + pvd.prior + arrhythmia.prior + copd + diabetes.prior + ulcer.prior + renal.prior +
rheuma.prior + cancer.prior + alcohol.prior, data=datM))

resp <- Surv(datF$depression.day, datF$depression)
res <- coxph(resp ~ med + age + townsend_quintile + sha1 + selfharm.prior + depression.prior + ihd.prior + stroke.prior
+ hf.prior + pvd.prior + arrhythmia.prior + copd + diabetes.prior + ulcer.prior + renal.prior + rheuma.prior + cancer.prior
+ alcohol.prior, data=datF)
summary(res)
summary(coxph(resp ~ relevel(med, ref="B") + age + townsend_quintile + sha1 + selfharm.prior + depression.prior +
ihd.prior + stroke.prior + hf.prior + pvd.prior + arrhythmia.prior + copd + diabetes.prior + ulcer.prior + renal.prior +
rheuma.prior + cancer.prior + alcohol.prior, data=datF))

### adjusted Cox proportional hazard model: arrhythmia in males and females separately

resp <- Surv(datM$arrhythmia.day, datM$arrhythmia)
```

```
res <- coxph(resp ~ med + age + townsend_quintile + sha1 + selfharm.prior + depression.prior + ihd.prior + stroke.prior
+ hf.prior + pvd.prior + arrhythmia.prior + copd + diabetes.prior + ulcer.prior + renal.prior + rheuma.prior + cancer.prior
+ alcohol.prior, data=datM)
summary(res)
summary(coxph(resp ~ relevel(med, ref="B") + age + townsend_quintile + sha1 + selfharm.prior + depression.prior +
ihd.prior + stroke.prior + hf.prior + pvd.prior + arrhythmia.prior + copd + diabetes.prior + ulcer.prior + renal.prior +
rheuma.prior + cancer.prior + alcohol.prior, data=datM))

resp <- Surv(datF$arrhythmia.day, datF$arrhythmia)
res <- coxph(resp ~ med + age + townsend_quintile + sha1 + selfharm.prior + depression.prior + ihd.prior + stroke.prior
+ hf.prior + pvd.prior + arrhythmia.prior + copd + diabetes.prior + ulcer.prior + renal.prior + rheuma.prior + cancer.prior
+ alcohol.prior, data=datF)
summary(res)
summary(coxph(resp ~ relevel(med, ref="B") + age + townsend_quintile + sha1 + selfharm.prior + depression.prior +
ihd.prior + stroke.prior + hf.prior + pvd.prior + arrhythmia.prior + copd + diabetes.prior + ulcer.prior + renal.prior +
rheuma.prior + cancer.prior + alcohol.prior, data=datF))

###3-month outcomes#####################################################################################
###3-month outcomes#####################################################################################
###3-month outcomes#####################################################################################

### number of patients and patient-years per drug group
table(dat$med)

sum(dat$selfharm.day.3m[dat$med == "N"])/365 #other way of coding: sum(dat[dat$med == "N","selfharm.day.3m"])
sum(dat$selfharm.day.3m[dat$med == "B"])/365
sum(dat$selfharm.day.3m[dat$med == "V"])/365
sum(dat$depression.day.3m[dat$med == "N"])/365
sum(dat$depression.day.3m[dat$med == "B"])/365
sum(dat$depression.day.3m[dat$med == "V"])/365
sum(dat$ihd.day.3m[dat$med == "N"])/365
sum(dat$ihd.day.3m[dat$med == "B"])/365
sum(dat$ihd.day.3m[dat$med == "V"])/365
sum(dat$stroke.day.3m[dat$med == "N"])/365
sum(dat$stroke.day.3m[dat$med == "B"])/365
sum(dat$stroke.day.3m[dat$med == "V"])/365
sum(dat$hf.day.3m[dat$med == "N"])/365
sum(dat$hf.day.3m[dat$med == "B"])/365
sum(dat$hf.day.3m[dat$med == "V"])/365
sum(dat$pvd.day.3m[dat$med == "N"])/365
sum(dat$pvd.day.3m[dat$med == "B"])/365
sum(dat$pvd.day.3m[dat$med == "V"])/365
sum(dat$arrhythmia.day.3m[dat$med == "N"])/365
sum(dat$arrhythmia.day.3m[dat$med == "B"])/365
sum(dat$arrhythmia.day.3m[dat$med == "V"])/365

### number and proportion of patients that experienced the specific event
table(dat$med, dat$selfharm.3m)
prop.table(table(dat$med, dat$selfharm.3m), margin=1)
table(dat$med, dat$depression.3m)
prop.table(table(dat$med, dat$depression.3m), margin=1)
table(dat$med, dat$ihd.3m)
prop.table(table(dat$med, dat$ihd.3m), margin=1)
table(dat$med, dat$stroke.3m)
prop.table(table(dat$med, dat$stroke.3m), margin=1)
table(dat$med, dat$hf.3m)
prop.table(table(dat$med, dat$hf.3m), margin=1)
```

```
table(dat$med, dat$pvd.3m)
prop.table(table(dat$med, dat$pvd.3m), margin=1)
table(dat$med, dat$arrhythmia.3m)
prop.table(table(dat$med, dat$arrhythmia.3m), margin=1)

###Survival analyses (3m)#########################################################################

### survival analysis and Kaplan-Meier curves: selfharm

resp <- Surv(dat$selfharm.day.3m, dat$selfharm.3m)

ylow <- .90 #lower y-axis limit for KM curve

res <- survfit(resp ~ med, data=dat)
print(res, print.rmean=TRUE)

png(filename="fig_KM_selfharm_3m.png", res=350, width=3000, height=2500, type="cairo") #to save as png file

plot(res, col=c("blue", "red", "green"), xlab="Days", ylab="Survival Proportion", xlim=c(0,93), ylim=c(ylow,1), xaxt="n")
axis(side=1, at=seq(0,90,20))
legend(x=3, y=ylow, legend=c("NRT Rx", "Bupropion", "Varenicline"), col=c("blue", "red", "green"), lty="solid", yjust=0,
bty="n")
title("Self-harm during 3 months follow-up")

dev.off()

### survival analysis and Kaplan-Meier curves: depression

resp <- Surv(dat$depression.day.3m, dat$depression.3m)

ylow <- .90 #lower y-axis limit for KM curve

res <- survfit(resp ~ med, data=dat)
print(res, print.rmean=TRUE)

png(filename="fig_KM_depression_3m.png", res=350, width=3000, height=2500, type="cairo") #to save as png file

plot(res, col=c("blue", "red", "green"), xlab="Days", ylab="Survival Proportion", xlim=c(0,93), ylim=c(ylow,1), xaxt="n")
axis(side=1, at=seq(0,90,20))
legend(x=3, y=ylow, legend=c("NRT Rx", "Bupropion", "Varenicline"), col=c("blue", "red", "green"), lty="solid", yjust=0,
bty="n")
title("Depression during 3 months follow-up")

dev.off()

### survival analysis and Kaplan-Meier curves: ihd

resp <- Surv(dat$ihd.day.3m, dat$ihd.3m)

ylow <- .90 #lower y-axis limit for KM curve

res <- survfit(resp ~ med, data=dat)
print(res, print.rmean=TRUE)

png(filename="fig_KM_ihd_3m.png", res=350, width=3000, height=2500, type="cairo") #to save as png file

plot(res, col=c("blue", "red", "green"), xlab="Days", ylab="Survival Proportion", xlim=c(0,93), ylim=c(ylow,1), xaxt="n")
```

```
axis(side=1, at=seq(0,90,20))
legend(x=3, y=ylow, legend=c("NRT Rx", "Bupropion", "Varenicline"), col=c("blue", "red", "green"), lty="solid", yjust=0, bty="n")
title("Ischaemic heart disease during 3 months follow-up")

dev.off()

### survival analysis and Kaplan-Meier curves: stroke

resp <- Surv(dat$stroke.day.3m, dat$stroke.3m)

ylow <- .90 #lower y-axis limit for KM curve

res <- survfit(resp ~ med, data=dat)
print(res, print.rmean=TRUE)

png(filename="fig_KM_stroke_3m.png", res=350, width=3000, height=2500, type="cairo") #to save as png file

plot(res, col=c("blue", "red", "green"), xlab="Days", ylab="Survival Proportion", xlim=c(0,93), ylim=c(ylow,1), xaxt="n")
axis(side=1, at=seq(0,90,20))
legend(x=3, y=ylow, legend=c("NRT Rx", "Bupropion", "Varenicline"), col=c("blue", "red", "green"), lty="solid", yjust=0, bty="n")
title("Stroke during 3 months follow-up")

dev.off()

### survival analysis and Kaplan-Meier curves: hf

resp <- Surv(dat$hf.day.3m, dat$hf.3m)

ylow <- .90 #lower y-axis limit for KM curve

res <- survfit(resp ~ med, data=dat)
print(res, print.rmean=TRUE)

png(filename="fig_KM_hf_3m.png", res=350, width=3000, height=2500, type="cairo") #to save as png file

plot(res, col=c("blue", "red", "green"), xlab="Days", ylab="Survival Proportion", xlim=c(0,93), ylim=c(ylow,1), xaxt="n")
axis(side=1, at=seq(0,90,20))
legend(x=3, y=ylow, legend=c("NRT Rx", "Bupropion", "Varenicline"), col=c("blue", "red", "green"), lty="solid", yjust=0, bty="n")
title("Heart failure during 3 months follow-up")

dev.off()

### survival analysis and Kaplan-Meier curves: pvd

resp <- Surv(dat$pvd.day.3m, dat$pvd.3m)

ylow <- .90 #lower y-axis limit for KM curve

res <- survfit(resp ~ med, data=dat)
print(res, print.rmean=TRUE)

png(filename="fig_KM_pvd_3m.png", res=350, width=3000, height=2500, type="cairo") #to save as png file

plot(res, col=c("blue", "red", "green"), xlab="Days", ylab="Survival Proportion", xlim=c(0,93), ylim=c(ylow,1), xaxt="n")
```

```
axis(side=1, at=seq(0,90,20))
legend(x=3, y=ylow, legend=c("NRT Rx", "Bupropion", "Varenicline"), col=c("blue", "red", "green"), lty="solid", yjust=0,
bty="n")
title("Peripheral vascular disease during 3 months follow-up")

dev.off()

### survival analysis and Kaplan-Meier curves: arrhythmia

resp <- Surv(dat$arrhythmia.day.3m, dat$arrhythmia.3m)

ylow <- .90 #lower y-axis limit for KM curve

res <- survfit(resp ~ med, data=dat)
print(res, print.rmean=TRUE)

png(filename="fig_KM_arrhythmia_3m.png", res=350, width=3000, height=2500, type="cairo") #to save as png file

plot(res, col=c("blue", "red", "green"), xlab="Days", ylab="Survival Proportion", xlim=c(0,93), ylim=c(ylow,1), xaxt="n")
axis(side=1, at=seq(0,90,20))
legend(x=3, y=ylow, legend=c("NRT Rx", "Bupropion", "Varenicline"), col=c("blue", "red", "green"), lty="solid", yjust=0,
bty="n")
title("Cardiac arrhythmia during 3 months follow-up")

dev.off()

### crude and adjusted Cox proportional hazard model, and test for interaction (med x sex): selfharm

resp <- Surv(dat$selfharm.day.3m, dat$selfharm.3m)

res <- coxph(resp ~ med, data=dat)
summary(res)
summary(coxph(resp ~ relevel(med, ref="B"), data=dat))
cox.zph(res)

res <- coxph(resp ~ med + age + sex + townsend_quintile + sha1 + selfharm.prior + depression.prior + ihd.prior +
stroke.prior + hf.prior + pvd.prior + arrhythmia.prior + copd + diabetes.prior + ulcer.prior + renal.prior + rheuma.prior +
cancer.prior + alcohol.prior, data=dat)
summary(res)
summary(coxph(resp ~ relevel(med, ref="B") + age + sex + townsend_quintile + sha1 + selfharm.prior + depression.prior
+ ihd.prior + stroke.prior + hf.prior + pvd.prior + arrhythmia.prior + copd + diabetes.prior + ulcer.prior + renal.prior +
rheuma.prior + cancer.prior + alcohol.prior, data=dat))

res1 <- coxph(resp ~ med*sex + age + townsend_quintile + sha1 + selfharm.prior + depression.prior + ihd.prior +
stroke.prior + hf.prior + pvd.prior + arrhythmia.prior + copd + diabetes.prior + ulcer.prior + renal.prior + rheuma.prior +
cancer.prior + alcohol.prior, data=dat)
anova(res1, res)
summary(res1)
summary(coxph(resp ~ med*relevel(sex, ref="male") + age + townsend_quintile + sha1 + selfharm.prior +
depression.prior + ihd.prior + stroke.prior + hf.prior + pvd.prior + arrhythmia.prior + copd + diabetes.prior + ulcer.prior
+ renal.prior + rheuma.prior + cancer.prior + alcohol.prior, data=dat))

### crude and adjusted Cox proportional hazard model, and test for interaction (med x sex): depression

resp <- Surv(dat$depression.day.3m, dat$depression.3m)

res <- coxph(resp ~ med, data=dat)
```

```
summary(res)
summary(coxph(resp ~ relevel(med, ref="B"), data=dat))
cox.zph(res)

res <- coxph(resp ~ med + age + sex + townsend_quintile + sha1 + selfharm.prior + depression.prior + ihd.prior +
stroke.prior + hf.prior + pvd.prior + arrhythmia.prior + copd + diabetes.prior + ulcer.prior + renal.prior + rheuma.prior +
cancer.prior + alcohol.prior, data=dat)
summary(res)
summary(coxph(resp ~ relevel(med, ref="B") + age + sex + townsend_quintile + sha1 + selfharm.prior + depression.prior
+ ihd.prior + stroke.prior + hf.prior + pvd.prior + arrhythmia.prior + copd + diabetes.prior + ulcer.prior + renal.prior +
rheuma.prior + cancer.prior + alcohol.prior, data=dat))

res1 <- coxph(resp ~ med*sex + age + townsend_quintile + sha1 + selfharm.prior + depression.prior + ihd.prior +
stroke.prior + hf.prior + pvd.prior + arrhythmia.prior + copd + diabetes.prior + ulcer.prior + renal.prior + rheuma.prior +
cancer.prior + alcohol.prior, data=dat)
anova(res1, res)
summary(res1)
summary(coxph(resp ~ med*relevel(sex, ref="male") + age + townsend_quintile + sha1 + selfharm.prior +
depression.prior + ihd.prior + stroke.prior + hf.prior + pvd.prior + arrhythmia.prior + copd + diabetes.prior + ulcer.prior
+ renal.prior + rheuma.prior + cancer.prior + alcohol.prior, data=dat))

### crude and adjusted Cox proportional hazard model, and test for interaction (med x sex): ihd

resp <- Surv(dat$ihd.day.3m, dat$ihd.3m)

res <- coxph(resp ~ med, data=dat)
summary(res)
summary(coxph(resp ~ relevel(med, ref="B"), data=dat))
cox.zph(res)

res <- coxph(resp ~ med + age + sex + townsend_quintile + sha1 + selfharm.prior + depression.prior + ihd.prior +
stroke.prior + hf.prior + pvd.prior + arrhythmia.prior + copd + diabetes.prior + ulcer.prior + renal.prior + rheuma.prior +
cancer.prior + alcohol.prior, data=dat)
summary(res)
summary(coxph(resp ~ relevel(med, ref="B") + age + sex + townsend_quintile + sha1 + selfharm.prior + depression.prior
+ ihd.prior + stroke.prior + hf.prior + pvd.prior + arrhythmia.prior + copd + diabetes.prior + ulcer.prior + renal.prior +
rheuma.prior + cancer.prior + alcohol.prior, data=dat))

res1 <- coxph(resp ~ med*sex + age + townsend_quintile + sha1 + selfharm.prior + depression.prior + ihd.prior +
stroke.prior + hf.prior + pvd.prior + arrhythmia.prior + copd + diabetes.prior + ulcer.prior + renal.prior + rheuma.prior +
cancer.prior + alcohol.prior, data=dat)
anova(res1, res)
summary(res1)
summary(coxph(resp ~ med*relevel(sex, ref="male") + age + townsend_quintile + sha1 + selfharm.prior +
depression.prior + ihd.prior + stroke.prior + hf.prior + pvd.prior + arrhythmia.prior + copd + diabetes.prior + ulcer.prior
+ renal.prior + rheuma.prior + cancer.prior + alcohol.prior, data=dat))

### crude and adjusted Cox proportional hazard model, and test for interaction (med x sex): stroke

resp <- Surv(dat$stroke.day.3m, dat$stroke.3m)

res <- coxph(resp ~ med, data=dat)
summary(res)
summary(coxph(resp ~ relevel(med, ref="B"), data=dat))
cox.zph(res)
```

```
res <- coxph(resp ~ med + age + sex + townsend_quintile + sha1 + selfharm.prior + depression.prior + ihd.prior +
stroke.prior + hf.prior + pvd.prior + arrhythmia.prior + copd + diabetes.prior + ulcer.prior + renal.prior + rheuma.prior +
cancer.prior + alcohol.prior, data=dat)
summary(res)
summary(coxph(resp ~ relevel(med, ref="B") + age + sex + townsend_quintile + sha1 + selfharm.prior + depression.prior
+ ihd.prior + stroke.prior + hf.prior + pvd.prior + arrhythmia.prior + copd + diabetes.prior + ulcer.prior + renal.prior +
rheuma.prior + cancer.prior + alcohol.prior, data=dat))

res1 <- coxph(resp ~ med*sex + age + townsend_quintile + sha1 + selfharm.prior + depression.prior + ihd.prior +
stroke.prior + hf.prior + pvd.prior + arrhythmia.prior + copd + diabetes.prior + ulcer.prior + renal.prior + rheuma.prior +
cancer.prior + alcohol.prior, data=dat)
anova(res1, res)
summary(res1)
summary(coxph(resp ~ med*relevel(sex, ref="male") + age + townsend_quintile + sha1 + selfharm.prior +
depression.prior + ihd.prior + stroke.prior + hf.prior + pvd.prior + arrhythmia.prior + copd + diabetes.prior + ulcer.prior
+ renal.prior + rheuma.prior + cancer.prior + alcohol.prior, data=dat))

### crude and adjusted Cox proportional hazard model, and test for interaction (med x sex): hf

resp <- Surv(dat$hf.day.3m, dat$hf.3m)

res <- coxph(resp ~ med, data=dat)
summary(res)
summary(coxph(resp ~ relevel(med, ref="B"), data=dat))
cox.zph(res)

res <- coxph(resp ~ med + age + sex + townsend_quintile + sha1 + selfharm.prior + depression.prior + ihd.prior +
stroke.prior + hf.prior + pvd.prior + arrhythmia.prior + copd + diabetes.prior + ulcer.prior + renal.prior + rheuma.prior +
cancer.prior + alcohol.prior, data=dat)
summary(res)
summary(coxph(resp ~ relevel(med, ref="B") + age + sex + townsend_quintile + sha1 + selfharm.prior + depression.prior
+ ihd.prior + stroke.prior + hf.prior + pvd.prior + arrhythmia.prior + copd + diabetes.prior + ulcer.prior + renal.prior +
rheuma.prior + cancer.prior + alcohol.prior, data=dat))

res1 <- coxph(resp ~ med*sex + age + townsend_quintile + sha1 + selfharm.prior + depression.prior + ihd.prior +
stroke.prior + hf.prior + pvd.prior + arrhythmia.prior + copd + diabetes.prior + ulcer.prior + renal.prior + rheuma.prior +
cancer.prior + alcohol.prior, data=dat)
anova(res1, res)
summary(res1)
summary(coxph(resp ~ med*relevel(sex, ref="male") + age + townsend_quintile + sha1 + selfharm.prior +
depression.prior + ihd.prior + stroke.prior + hf.prior + pvd.prior + arrhythmia.prior + copd + diabetes.prior + ulcer.prior
+ renal.prior + rheuma.prior + cancer.prior + alcohol.prior, data=dat))

### crude and adjusted Cox proportional hazard model, and test for interaction (med x sex): pvd

resp <- Surv(dat$pvd.day.3m, dat$pvd.3m)

res <- coxph(resp ~ med, data=dat)
summary(res)
summary(coxph(resp ~ relevel(med, ref="B"), data=dat))
cox.zph(res)

res <- coxph(resp ~ med + age + sex + townsend_quintile + sha1 + selfharm.prior + depression.prior + ihd.prior +
stroke.prior + hf.prior + pvd.prior + arrhythmia.prior + copd + diabetes.prior + ulcer.prior + renal.prior + rheuma.prior +
cancer.prior + alcohol.prior, data=dat)
summary(res)
```

```
summary(coxph(resp ~ relevel(med, ref="B") + age + sex + townsend_quintile + sha1 + selfharm.prior + depression.prior
+ ihd.prior + stroke.prior + hf.prior + pvd.prior + arrhythmia.prior + copd + diabetes.prior + ulcer.prior + renal.prior +
rheuma.prior + cancer.prior + alcohol.prior, data=dat))

res1 <- coxph(resp ~ med*sex + age + townsend_quintile + sha1 + selfharm.prior + depression.prior + ihd.prior +
stroke.prior + hf.prior + pvd.prior + arrhythmia.prior + copd + diabetes.prior + ulcer.prior + renal.prior + rheuma.prior +
cancer.prior + alcohol.prior, data=dat)
anova(res1, res)
summary(res1)
summary(coxph(resp ~ med*relevel(sex, ref="male") + age + townsend_quintile + sha1 + selfharm.prior +
depression.prior + ihd.prior + stroke.prior + hf.prior + pvd.prior + arrhythmia.prior + copd + diabetes.prior + ulcer.prior
+ renal.prior + rheuma.prior + cancer.prior + alcohol.prior, data=dat))

### crude and adjusted Cox proportional hazard model, and test for interaction (med x sex): arrhythmia

resp <- Surv(dat$arrhythmia.day.3m, dat$arrhythmia.3m)

res <- coxph(resp ~ med, data=dat)
summary(res)
summary(coxph(resp ~ relevel(med, ref="B"), data=dat))
cox.zph(res)

res <- coxph(resp ~ med + age + sex + townsend_quintile + sha1 + selfharm.prior + depression.prior + ihd.prior +
stroke.prior + hf.prior + pvd.prior + arrhythmia.prior + copd + diabetes.prior + ulcer.prior + renal.prior + rheuma.prior +
cancer.prior + alcohol.prior, data=dat)
summary(res)
summary(coxph(resp ~ relevel(med, ref="B") + age + sex + townsend_quintile + sha1 + selfharm.prior + depression.prior
+ ihd.prior + stroke.prior + hf.prior + pvd.prior + arrhythmia.prior + copd + diabetes.prior + ulcer.prior + renal.prior +
rheuma.prior + cancer.prior + alcohol.prior, data=dat))

res1 <- coxph(resp ~ med*sex + age + townsend_quintile + sha1 + selfharm.prior + depression.prior + ihd.prior +
stroke.prior + hf.prior + pvd.prior + arrhythmia.prior + copd + diabetes.prior + ulcer.prior + renal.prior + rheuma.prior +
cancer.prior + alcohol.prior, data=dat)
anova(res1, res)
summary(res1)
summary(coxph(resp ~ med*relevel(sex, ref="male") + age + townsend_quintile + sha1 + selfharm.prior +
depression.prior + ihd.prior + stroke.prior + hf.prior + pvd.prior + arrhythmia.prior + copd + diabetes.prior + ulcer.prior
+ renal.prior + rheuma.prior + cancer.prior + alcohol.prior, data=dat))

###Propensity scores (3m)#########################################################################

### propensity score analysis for N vs. B - step 1: trim and match 1:1

datNB <- subset(dat, med == "N" | med == "B")
datNB$med <- factor(datNB$med, levels=c("N","B"))
str(datNB)
table(datNB$med)

datNB$medB <- ifelse(datNB$med == "B", 1, 0)
res <- glm(medB ~ age + sex + townsend_quintile + sha1 + selfharm.prior + depression.prior + ihd.prior + stroke.prior +
hf.prior + pvd.prior + arrhythmia.prior + copd + diabetes.prior + ulcer.prior + renal.prior + rheuma.prior + cancer.prior +
alcohol.prior, family=binomial, data=datNB)
preds <- predict(res, type="response") #preds = predicted probabilities = propensity scores
by(preds, datNB$med, summary)
nrow(datNB)
table(datNB$med)
```

```
### ROC curve and AUC
pred <- prediction(preds, datNB$medB)
perf <- performance(pred, measure = "tpr", x.measure = "fpr")
plot(perf, lwd=2)
abline(a=0, b=1, lty="dotted")
performance(pred, measure="auc")@"y.values"[[1]]

### trim away the highest propensity scores of the N group and the lowest scores of the B group
sel1 <- datNB$med == "B" | (datNB$med == "N" & preds < quantile(preds[datNB$med == "N"], .975))
sel2 <- datNB$med == "N" | (datNB$med == "B" & preds > quantile(preds[datNB$med == "B"], .025))
datNB <- datNB[sel1 & sel2,]
preds <- preds[sel1 & sel2]

by(preds, datNB$med, summary)
nrow(datNB)

datNB$preds <- preds

dat.temp <- datNB[,c("medB","preds")]
res <- matchit(medB ~ preds, data=dat.temp)

datNB <- datNB[is.element(rownames(datNB), c(row.names(res$match.matrix), c(res$match.matrix))),]

table(datNB$med)
by(datNB$preds, datNB$med, summary)

### propensity score analysis for N vs. B - step 2: Cox models for all events

resp <- Surv(datNB$selfharm.day.3m, datNB$selfharm.3m)
res <- coxph(resp ~ med, data=datNB)
summary(res)
cox.zph(res)

resp <- Surv(datNB$depression.day.3m, datNB$depression.3m)
res <- coxph(resp ~ med, data=datNB)
summary(res)
cox.zph(res)

resp <- Surv(datNB$ihd.day.3m, datNB$ihd.3m)
res <- coxph(resp ~ med, data=datNB)
summary(res)
cox.zph(res)

resp <- Surv(datNB$stroke.day.3m, datNB$stroke.3m)
res <- coxph(resp ~ med, data=datNB)
summary(res)
cox.zph(res)

resp <- Surv(datNB$hf.day.3m, datNB$hf.3m)
res <- coxph(resp ~ med, data=datNB)
summary(res)
cox.zph(res)

resp <- Surv(datNB$pvd.day.3m, datNB$pvd.3m)
res <- coxph(resp ~ med, data=datNB)
summary(res)
cox.zph(res)
```

```
resp <- Surv(datNB$arrhythmia.day.3m, datNB$arrhythmia.3m)
res <- coxph(resp ~ med, data=datNB)
summary(res)
cox.zph(res)

### propensity score analysis for N vs. V - step 1: trim and match 1:1

datVB <- subset(dat, med == "N" | med == "V")
datVB$med <- factor(datVB$med, levels=c("N","V"))
str(datVB)
nrow(datVB)
table(datVB$med)

datVB$medV <- ifelse(datVB$med == "V", 1, 0)
res <- glm(medV ~ age + sex + townsend_quintile + sha1 + selfharm.prior + depression.prior + ihd.prior + stroke.prior +
hf.prior + pvd.prior + arrhythmia.prior + copd + diabetes.prior + ulcer.prior + renal.prior + rheuma.prior + cancer.prior +
alcohol.prior, family=binomial, data=datVB)
preds <- predict(res, type="response") #preds = predicted probabilities = propensity scores
by(preds, datVB$med, summary)

### ROC curve and AUC
pred <- prediction(preds, datVB$medV)
perf <- performance(pred, measure = "tpr", x.measure = "fpr")
plot(perf, lwd=2)
abline(a=0, b=1, lty="dotted")
performance(pred, measure="auc")@"y.values"[[1]]

### trim away the highest propensity scores of the N group and the lowest scores of the B group
sel1 <- datVB$med == "V" | (datVB$med == "N" & preds < quantile(preds[datVB$med == "N"], .975))
sel2 <- datVB$med == "N" | (datVB$med == "V" & preds > quantile(preds[datVB$med == "V"], .025))
datVB <- datVB[sel1 & sel2,]
preds <- preds[sel1 & sel2]

by(preds, datVB$med, summary)
nrow(datVB)

datVB$preds <- preds

dat.temp <- datVB[,c("medV","preds")]
res <- matchit(medV ~ preds, data=dat.temp)

datVB <- datVB[is.element(rownames(datVB), c(row.names(res$match.matrix), c(res$match.matrix))),]

table(datVB$med)
by(datVB$preds, datVB$med, summary)

### propensity score analysis for N vs. V - step 2: Cox models for all events

resp <- Surv(datVB$selfharm.day.3m, datVB$selfharm.3m)
res <- coxph(resp ~ med, data=datVB)
summary(res)
cox.zph(res)

resp <- Surv(datVB$depression.day.3m, datVB$depression.3m)
res <- coxph(resp ~ med, data=datVB)
summary(res)
```

```
cox.zph(res)

resp <- Surv(datVB$ihd.day.3m, datVB$ihd.3m)
res <- coxph(resp ~ med, data=datVB)
summary(res)
cox.zph(res)

resp <- Surv(datVB$stroke.day.3m, datVB$stroke.3m)
res <- coxph(resp ~ med, data=datVB)
summary(res)
cox.zph(res)

resp <- Surv(datVB$hf.day.3m, datVB$hf.3m)
res <- coxph(resp ~ med, data=datVB)
summary(res)
cox.zph(res)

resp <- Surv(datVB$pvd.day.3m, datVB$pvd.3m)
res <- coxph(resp ~ med, data=datVB)
summary(res)
cox.zph(res)

resp <- Surv(datVB$arrhythmia.day.3m, datVB$arrhythmia.3m)
res <- coxph(resp ~ med, data=datVB)
summary(res)
cox.zph(res)

###### HR and CI bounds adjusted for an unmeasured confounder (based on Lin et al., 1998)

### observed HR and CI bounds: self-harm
HR    <- 0.56
CI.LB <- 0.46
CI.UB <- 0.68

### HR for the unmeasured confounder: self-harm
HRu <- 3

p1 <- p0 <- seq(0, 1, by=.1)
adj.fun <- function(p1,p0,R) {
  A <- (HRu * p1 + (1-p1)) / (HRu * p0 + (1-p0))
  R/A
}

### adjusted hazard ratio: self-harm
round(outer(p1, p0, FUN=adj.fun, R=HR), 2)

### adjusted lower and upper CI bounds: self-harm
round(outer(p1, p0, FUN=adj.fun, R=CI.LB), 2)
round(outer(p1, p0, FUN=adj.fun, R=CI.UB), 2)

###THE END#####################################################################################
```
